# The effect of a lateral alkyloxy chain on the ferroelectric nematic phase†

**DOI:** 10.1039/d2ra05628c

**Published:** 2022-10-14

**Authors:** Ewan Cruickshank, Rebecca Walker, John M. D. Storey, Corrie T. Imrie

**Affiliations:** Department of Chemistry, University of Aberdeen Old Aberdeen AB24 3UE UK ewan.cruickshank2@abdn.ac.uk

## Abstract

The synthesis and characterisation of two series of low molar mass liquid crystals, the 4-[(4-nitrophenoxy)carbonyl]phenyl 2-alkoxy-4-methoxybenzoates (series 5-*m*) and the 4-[(3-fluoro-4-nitrophenoxy)carbonyl]phenyl 2-alkoxy-4-methoxybenzoates (series 6-*m*) are reported in order to explore the effects of a lateral alkyloxy chain on the formation and stability of the recently discovered ferroelectric nematic phase. In both series *m*, the number of carbon atoms in the lateral chain, is varied from one to nine. The two series differ by the addition of a fluorine substituent in the 6-*m* series. 5-1 is the extensively studied ferroelectric nematogen RM734. All the members of the 5-*m* series exhibited both a conventional nematic, N, and ferroelectric nematic, N_F_, phase, whereas all the members of the 6-*m* series exhibit a direct N_F_–I transition with the exception of 6-1 that also exhibits a N phase. The replacement of a hydrogen atom by a fluorine atom reduces the nematic–isotropic transition temperature, *T*_NI_, whereas the ferroelectric nematic–nematic, or isotropic, transition temperature, *T*_N_F_N/I_, increases. This is interpreted in terms of the reduced structural anisotropy associated with the larger fluorine atom whereas the increase in the stability of the N_F_ phase reflects changes in polarity and polarizability. The dependence of *T*_NI_ and *T*_N_F_N/I_ on *m* in both series is similar, and these initially decrease on increasing *m* but converge to limiting values on further increasing *m*. This suggests that the lateral alkyloxy chain may adopt conformations in which it lies along the major axis of the mesogenic unit.

## Abstract 2

Properties of two series of ferronematogens are discussed to understand the influence of lateral bulk on the ferroelectric nematic phase.

## Graphical abstract

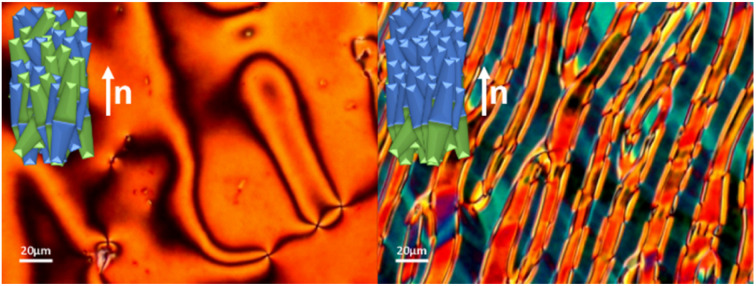

## Introduction

**Fig. 1:**
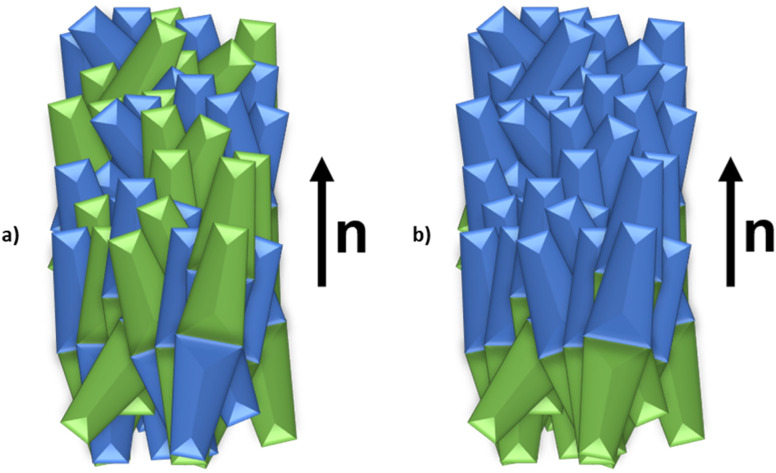
Schematic representations of the (a) conventional nematic, N, phase and (b) ferroelectric nematic, N_F_, phase.

The conventional nematic phase, N, is the least ordered liquid crystal phase and technologically the most important, underpinning the multi-billion-dollar LCD sector. In the N phase, the rod-like molecules align more or less in a common direction known as the director, represented by the unit vector ***n*** (Fig. 1(a)), whereas their centres of mass are distributed randomly, and so the N phase may be described as an orientationally ordered fluid. The director has inversion symmetry, *i.e.***n** = −**n**, and so the N phase is non-polar. One of the earliest attempts to develop a mathematical theory of liquid crystals was made by Born in which he assumed that the constituent molecules possessed a dipole moment and interactions between these drove the formation of the liquid crystalline phase.^1^ The underlying assumption was quickly proved to be incorrect, and non-polar molecules shown to exhibit liquid crystallinity. Indeed, as noted in a history of liquid crystals science, Born’s theory ‘has been relegated to the status of a historical footnote’.^2^ In principle, however, there was no fundamental reason why a nematic phase composed of polar molecules should not exhibit ferroelectric ordering, *i.e.***n** ≠ −**n**, (Fig. 1(b)), and this had been predicted by theoretical modelling.^3,4^ The ferroelectric nematic, N_F_, phase was not observed experimentally, however, until 2017 when a new type of nematic phase exhibiting ferroelectric properties was independently reported by Mandle *et al.* and Nishikawa *et al.* in the materials RM734 and DIO, respectively.^5,6^ This nematic phase was later assigned as the ferroelectric nematic phase, N_F_.^7^ The discovery of the N_F_ phase is of huge fundamental and technological significance, not least because it has a very high response sensitivity to electric fields with the application potential to dramatically improve the performance of LCDs. Indeed, the N_F_ phase is arguably the hottest topic in the science and technology of liquid crystals, elevating Born’s work from the status of a footnote.^8–23^

**Fig. 2:**
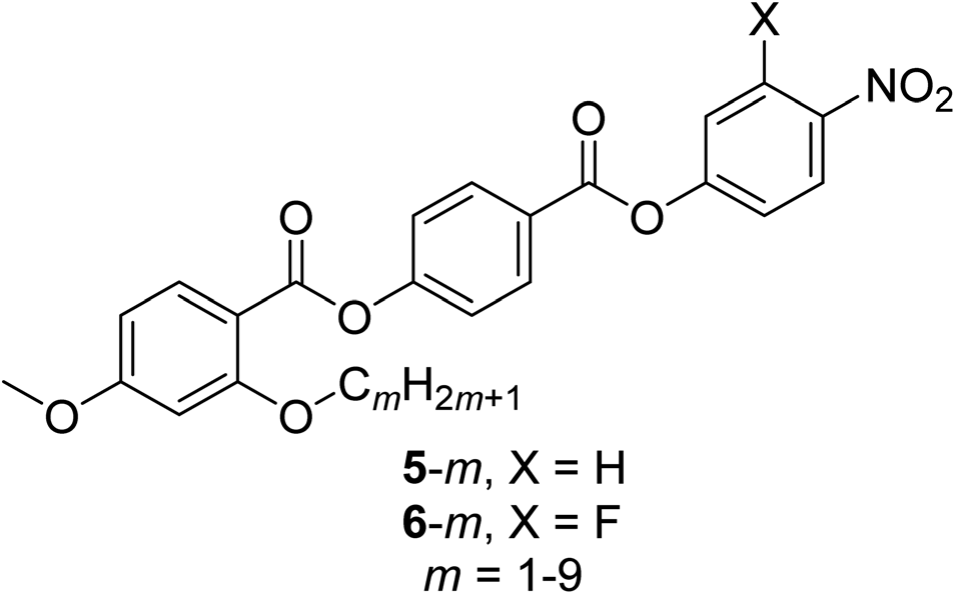
The structures of the 5-*m* and 6-*m* series where *m* refers to the number of carbon atoms in the lateral alkyloxy chain. 5-1 is the extensively studied ferroelectric nematogen RM734.^5^

Presently there are relatively few molecules known to exhibit the N_F_ phase, but these appear to have some common structural features thought to be important in driving the formation of the phase. The most critical molecular parameter appears to be a large dipole along the major axis of the molecule, and it has been suggested that this must be greater than 9 D.^24^ A second structural prerequisite is some degree of lateral steric bulk such that the molecule has, what may be described as, a wedge shape.^14,16^ Indeed, these empirical observations are consistent with computer simulations of tapered particles having a longitudinal dipole moment interacting within a generalised Gay-Berne type attractive-repulsive potential that showed a N_F_ phase.^25^ In order to establish and better understand the relationships between molecular structure and the formation of the N_F_ phase, it is critical that the library of materials which are known to exhibit the phase is expanded. In particular, a direct N_F_–I transition has been observed only in a very limited number of molecules.^23,26–29^ Using RM734 as a structural template, here we report the transitional properties of two series of ferroelectric nematogens containing a lateral alkyloxy chain, the 4-[(4-nitrophenoxy)carbonyl]phenyl 2-alkoxy-4-methoxybenzoates (5-*m*) and the 4-[(3-fluoro-4-nitrophenoxy)carbonyl]phenyl 2-alkoxy-4-methoxybenzoates (6-*m*) (Fig. 2). The length of this lateral chain has been varied to explore the effect of changing the shape of the molecule on the formation of the N_F_ phase. The two series differ by a fluorine atom on the nitrophenyl terminal ring, and this allows the effect of changing molecular polarity and polarizability to be considered. We note that of the eighteen ferroelectric nematogens described here, eight have been reported previously.^5,24,29^

## Experimental

### Synthesis

**Scheme 1:**
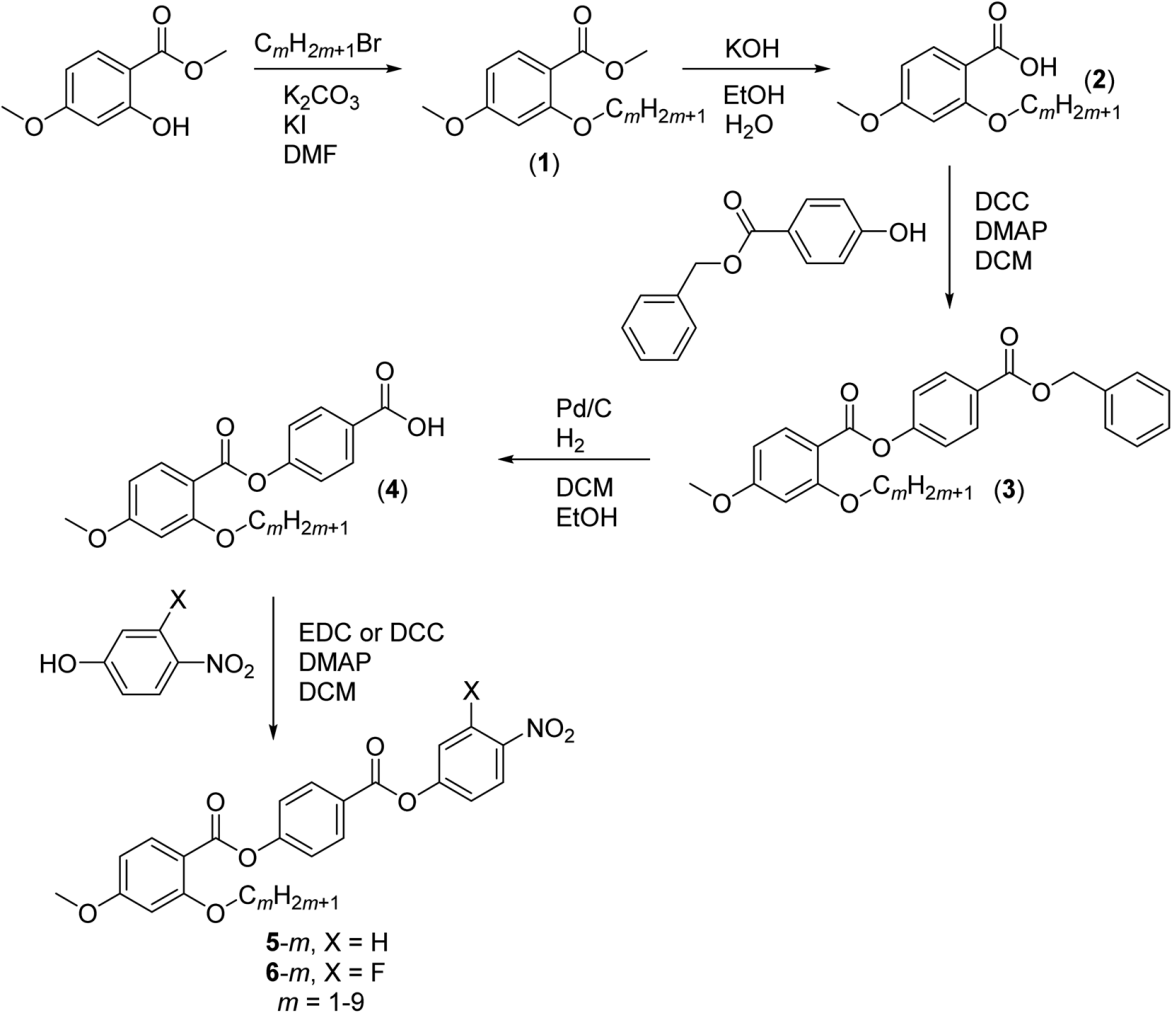
Synthesis of series 5-*m* and 6-*m*.

The synthetic route used to prepare the 5-*m* and 6-*m* series is shown in Scheme 1. A detailed description of the preparation of both series, including the structural characterisation data for all intermediates and final products, is provided in the ESI.†

### Optical studies

Phase characterisation was performed by polarised light microscopy, using an Olympus BH2 polarising light microscope equipped with a Linkam TMS 92 hot stage. The untreated glass slides were 0.17 mm thickness while the planar aligned cells were purchased from INSTEC with a cell thickness between 2.9 and 3.5 μm, and an ITO conducting layer.

### Differential scanning calorimetry

The phase behaviour of the materials was studied by differential scanning calorimetry performed using a Mettler Toledo DSC1 or DSC3 differential scanning calorimeter equipped with TSO 801RO sample robots and calibrated using indium and zinc standards. Heating and cooling rates were 10 °C min^−1^, with a 3 min isotherm between either heating or cooling, and all samples were measured under a nitrogen atmosphere. Transition temperatures and associated enthalpy changes were extracted from the heating traces unless otherwise noted.

### Molecular modelling

The geometric parameters of the 5-*m* and 6-*m* series were obtained using quantum mechanical DFT calculations with Gaussian 09 software.^30^ Optimisation of the molecular structures was carried out at the B3LYP/6-31G(d) level of theory. Visualizations of electronic surfaces were generated from the optimised geometries using the GaussView 5 software, and visualizations of the space-filling and ball-and-stick models were produced post-optimisation using the QuteMol package.^31^

## Results and discussion

The transitional properties of the 5-*m* series are listed in Table 1. We have previously reported the behaviour of 5-1 ^27^ and this was found to be in excellent agreement with that reported elsewhere.^5,7^ The transition temperatures for *m* = 2, 3, 5 and 6 were also found to be in excellent agreement with those reported in the literature.^24,29^ The data for 5-4 ^29^ has been reported previously. All the members of the 5-*m* series showed N_F_ and N phases. Both phases were monotropic for each homologue with the exception of 5-1 that showed an enantiotropic N phase.

**Table 1:** Transition temperatures and associated entropy changes for the 5-*m* series

*m*	*T*_Cr-_/°C	*T*_N_F_N_/°C	*T*_NI_/°C	Δ*S*_Cr-_/*R*	Δ*S*_N_F_N_/*R*	Δ*S*_NI_/*R*
1	139	131^a^	188	10.0	0.18^a^	0.16
2	159	106^a^	131^a^	13.5	0.14^a^	0.083^a^
3	147	85^a^	97^a^	12.5	0.30^a^	0.11^a^
4^c^	141	65^a^	75^a^	13.2	0.30^a^	0.097^a^
5	132	54^a^	61^a^	13.2	0.21^a^	0.069^a^
6	101	44^a^	50^a^	11.7	0.27^a^	0.15^a^
7	110	44^b^	50^b^	12.9	—	—
8	123	44^b^	51^b^	12.8	—	—
9	117	42^b^	49^b^	12.5	—	—

a Values extracted from DSC cooling traces.

b Measured using the polarised light microscope.

c Taken from Pociecha *et al.*^29^

**Fig. 3:**
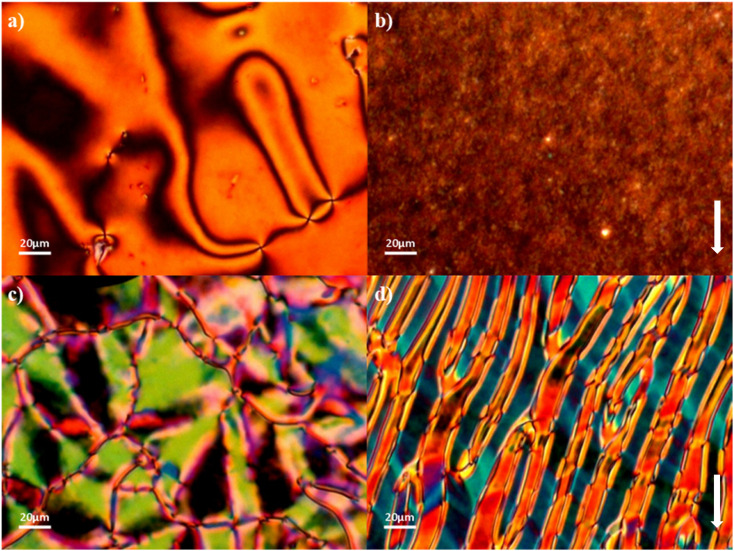
Optical textures observed for 5-2: (a) schlieren texture of the N phase between untreated glass slides (*T* = 130 °C); (b) uniform texture of the N phase in a planar aligned cell (*T* = 125 °C); (c) domains of differing birefringence with boundaries in the N_F_ phase between untreated glass slides (*T* = 102 °C); (d) banded texture of the N_F_ phase in a planar aligned cell (*T* = 100 °C). The arrow indicates the alignment direction.

The N_F_ and N phases were assigned on the basis of the optical textures observed using polarised optical microscopy. Specifically, the N phases were assigned from their characteristic schlieren textures containing two- and four-point brush defects which flashed when subjected to mechanical stress when viewed between untreated glass slides, and a uniform texture seen in planar aligned cells (Fig. 3(a) and (b), respectively). The scaled entropy changes associated with the N–I transition are consistent with this assignment, although the values are lower than normally observed and this may reflect the increased biaxiality of these compounds compared to that of conventional rod-like low molar mass mesogens.^32^ Cooling the N phase into the N_F_ phase sees the emergence of additional defects corresponding to domain boundaries dividing regions of different orientations of the director and hence differing polarizations (Fig. 3(c)). This texture flashes under mechanical stress and retains the fluidity of the N phase. In addition to the emergence of these domain boundaries at the N–N_F_ transition, there is also a distinct change in birefringence. This has been referred to as a banded texture,^24,28^ and is particularly distinct when viewed in a planar aligned cell, and appears to be characteristic of the N_F_ phase^27,28^ (Fig. 3(d)). The formation of these domains is thought to be driven by director splay deformations which are necessary to connect opposite polarisation vectors on the lower and upper cell surfaces.^27^

**Fig. 4:**
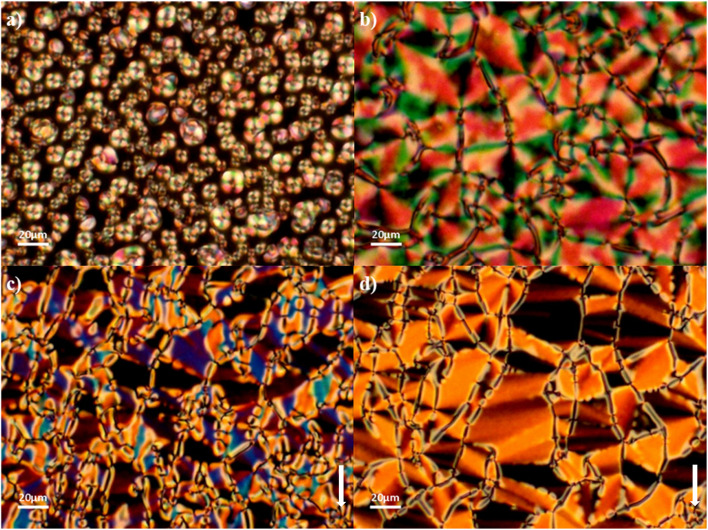
Optical textures observed for 6-2: when viewed between untreated glass slides, (a) growth of droplets at the I–N_F_ transition (*T* = 109 °C), and (b) domains of differing birefringence separated by boundaries in the N_F_ phase (*T* = 106 °C); when viewed in a planar aligned cell (c) banded texture of the N_F_ phase (*T* = 105 °C), and (d) banded texture with larger domains in the N_F_ phase (*T* = 101 °C). The arrow indicates the alignment direction.

The transitional properties of the 6-*m* series are listed in Table 2. The data for 6-1 (ref. 5) and 6-6 (ref. 29) have been reported previously. All the homologues showed a monotropic N_F_ phase and a direct N_F_–I phase transition with the single exception of 6-1 (ref. 5) which showed a monotropic conventional N phase preceding the N_F_ phase. The I–N_F_ transition when viewed between untreated glass slides is accompanied by the formation and growth of droplets on cooling (Fig. 4(a)) as described elsewhere.^26,28^ These droplets coalesce giving a texture (Fig. 4(b)) much like that seen between untreated glass slides for the 5-*m* series (Fig. 3(c)). Again, the separation of domains by distinct boundary lines to give the so-called banded texture is particularly evident when viewed in planar aligned cells (Fig. 4(c) and (d)). In many cases the N_F_ phase could be supercooled to room temperature without crystallisation. The scaled entropy changes associated with the N_F_–I transition for the 6-*m* series (Table 2) are considerably larger than the values of Δ*S*_NI_/*R* seen for the 5-*m* series (Table 1). This presumably reflects the additional entropic contribution associated with the ordering of the dipoles.^27^

**Table 2:** Transition temperatures and associated entropy changes for the 6-*m* series

*m*	*T*_Cr-_/°C	*T*_N_F_N_/°C	*T*_NI_/°C	Δ*S*_Cr-_/*R*	Δ*S*_N_F_N_/*R*	Δ*S*_NI_/*R*
		*T_N_F_I_/°C			*Δ*S*_N_F_I_/*R*	
1^b^	178	138^a^	154^a^	13.4	0.47^a^	0.14^a^
2	161	*109^a^	—	11.9	*0.65^a^	—
3	129	*88^a^	—	12.5	*0.84^a^	—
4	119	*69^a^	—	12.4	*0.77^a^	—
5	105	*56^a^	—	7.9	*0.53^a^	—
6^c^	77	*48^a^	—	13.3	*0.80^a^	—
7	93	*47^a^	—	12.5	*0.88^a^	—
8	111	*48^a^	—	14.4	*0.85^a^	—
9	105	*46^a^	—	14.5	*0.90^a^	—

a Values extracted from DSC cooling traces.

b Extracted from Mandle *et al.*^5^

c Taken from Pociecha *et al*.^29^

**Fig. 5:**
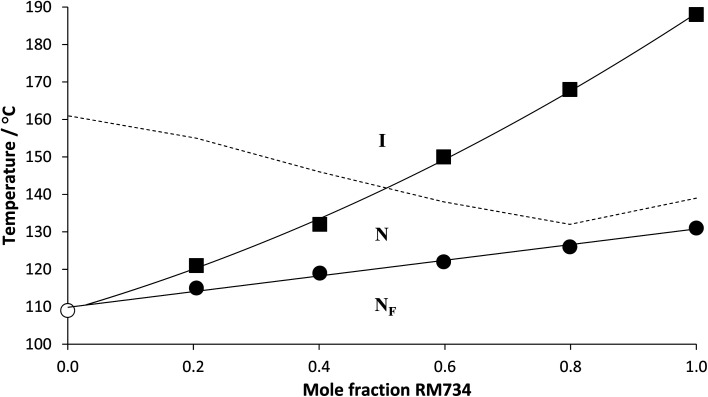
Phase diagram constructed using binary mixtures of 6-2 and 5-1 (RM734). Squares denote *T*_NI_, filled circles *T*_N_F_N_, the open circle *T*_N_F_I_ and the broken line connects the melting points. The solid lines indicate trend lines drawn for *T*_NI_ and *T*_N_F_N/I_.

**Fig. 6:**
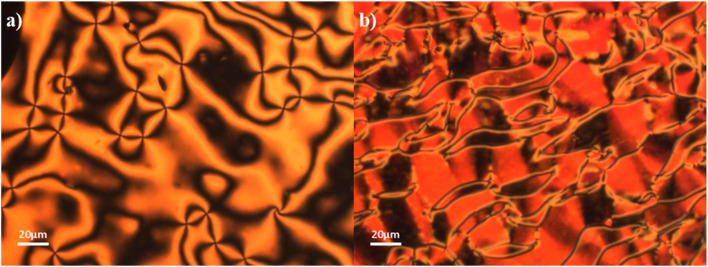
Optical textures observed for the 60:40 mol% 6-2:5-1 (RM734) mixture: (a) schlieren texture of the N phase between untreated glass slides (*T* = 125 °C); (b) banded texture of the N_F_ phase between untreated glass slides (*T* = 118 °C).

We note that in a previous study the assignment of the N_F_ phase for members of both series was confirmed using dielectric spectroscopy and second harmonic generation measurements.^29^ To further confirm the assignment of the N_F_ phase here, however, a phase diagram was constructed using binary mixtures of 6-2 and the well-characterised ferroelectric nematogen, 5-1 (RM734) (Fig. 5). All the mixtures studied exhibited two distinct liquid crystalline phases, the higher temperature phase was assigned as the conventional N phase on the basis of the observation of characteristic schlieren textures when viewed between two untreated glass slides (Fig. 6(a)). On cooling the nematic phase, a distinct shift in the sample birefringence was seen and a banded texture developed (Fig. 6(b)) much like that described earlier for the 5-*m* and 6-*m* series and assigned as the N_F_ phase. Both the N_F_–N and N–I transition temperatures of 5-1 decrease on the addition of 6-2 but the value of *T*_NI_ falls more rapidly. The trendlines associated with *T*_N_F_N_ and *T*_NI_ intersect at around 0.05 mol fraction of 5-1, and this is entirely consistent with the observation of a N_F_–I transition for 6-2. It is clear that the stability of the N phase is more sensitive to increased steric bulk than the N_F_ phase, and we will return to this theme later.

**Fig. 7:**
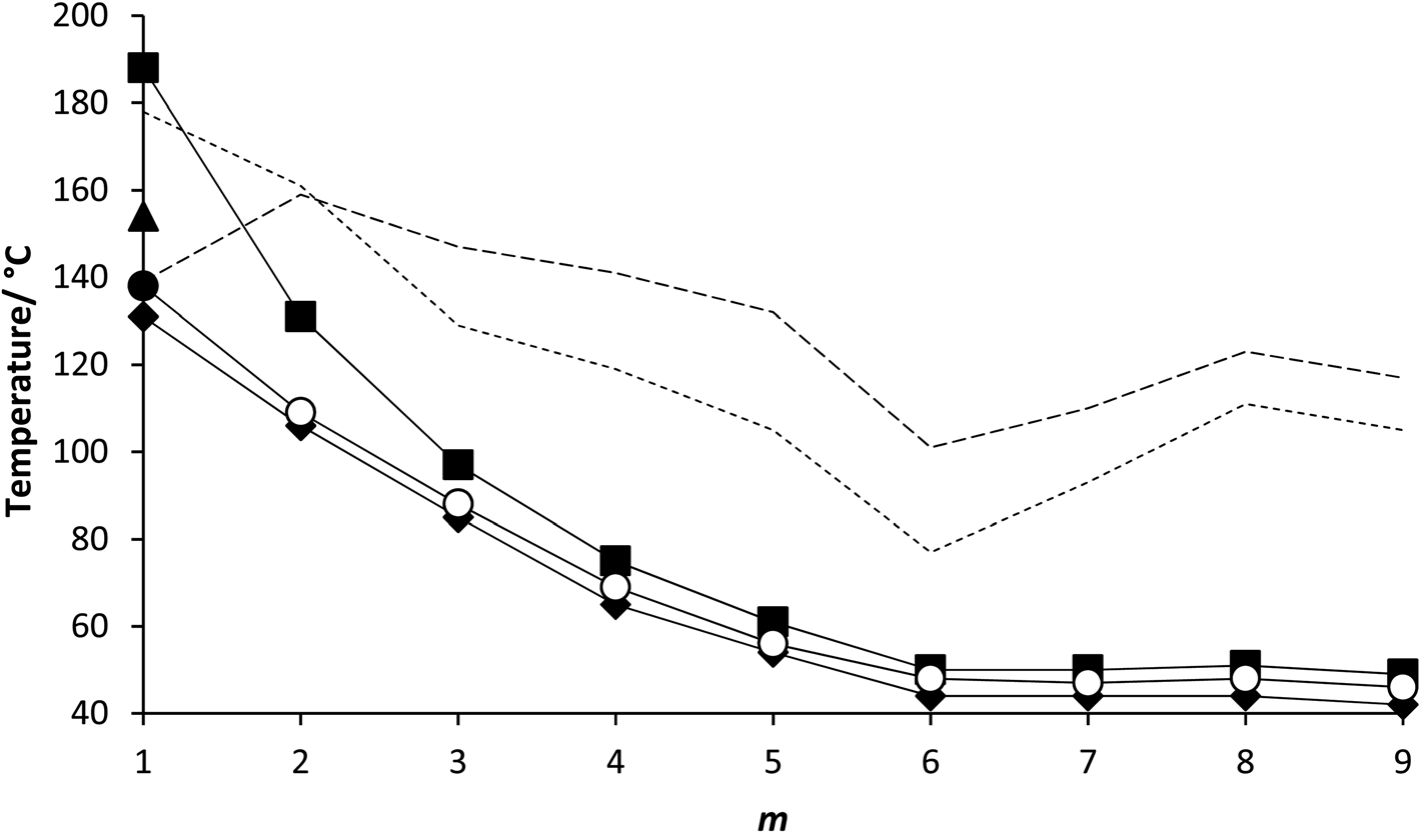
Dependence of the transition temperatures on the number of carbon atoms in the lateral alkyloxy chain, *m*, for the 5-*m* and 6-*m* series. For the 5-*m* series, the values of *T*_NI_ are represented by filled squares and *T*_N_F_N_ by filled diamonds. For the 6-*m* series, the value of *T*_NI_ is represented by a filled triangle, *T*_N_F_N_ by a filled circle and *T*_N_F_I_ by open circles. The melting points of the 5-*m* series are connected by the broken line with long dashes and for the 6-*m* series with short dashes.

**Fig. 8:**
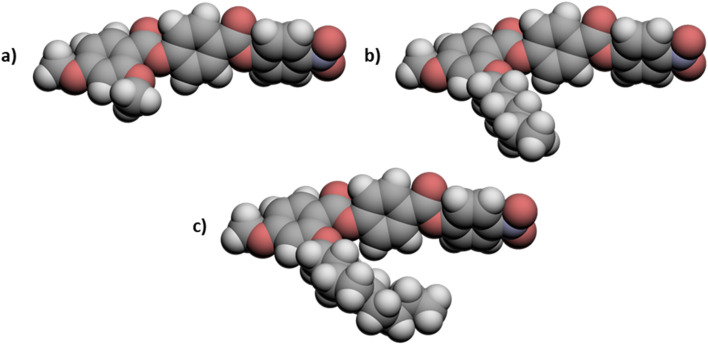
A comparison of the molecular shapes for members of the 5-*m* series: (a) 5-1, (b) 5-6 and (c) 5-9. In (c) the lateral chain contains gauche linkages causing it to lie along the molecular long axis, and the molecular breadth becomes insensitive to chain length.

**Fig. 9:**
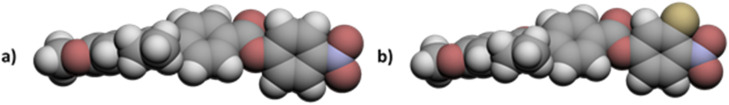
A comparison of the molecular shapes of (a) 5-2 and (b) 6-2.

The transition temperatures shown by the 5-*m* and 6-*m* series are compared in Fig. 7. The melting points of neither series show a regular dependence on varying *m* although those of the 5-*m* series are consistently higher. This suggests that the addition of the fluorine substituent reduces the packing efficiency of the molecules in the crystal phase. The values of *T*_NI_ for the 5-*m* series initially decrease with increasing *m* and converge to a limiting value on further increasing *m*. Similar behaviour was first reported for low molar mass mesogens containing a lateral alkyl chain by Weissflog and Demus,^33,34^ and thought to imply that the lateral alkyl chain adopts conformations in which it lies parallel to the molecular axis. Thus, initially increasing *m* decreases the length to breadth ratio of the molecule (Fig. 8(a) and (b)) and hence, *T*_NI_ falls. Above a given value of *m*, however, the chain extends along the molecular axis and hence the molecular breadth is constant over a range of chain lengths (Fig. 8(c)). It has been suggested, however, that this pattern of behaviour may be understood without invoking special conformations of the chain which confines it to lie parallel to the major axis of the mesogenic core but instead reflects that in these three-ring systems the dominant effect of increasing *m* is to dilute the interactions between the cores.^35,36^ It is possible that the nematic field forces the chain to adopt conformations in which it lies along the principal molecular axis, but such an assumption is not necessary to understand the dependence of *T*_NI_ on *m*. We have seen that only one member of the 6-*m* series shows the nematic phase, 6-1, and the addition of the fluorine substituent has reduced *T*_NI_ by 34 °C compared to that of 5-1. This may be attributed to the reduction in structural anisotropy on replacing a hydrogen atom by the larger fluorine atom (Fig. 9), and the magnitude of the decrease reflects the size of the substituent irrespective of its other properties such as polarizability and polarity.

**Fig. 10:**
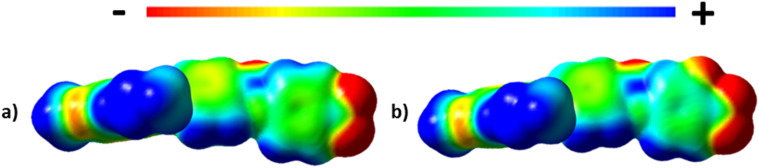
The electrostatic potential surfaces of (a) 5-2 and (b) 6-2.

The values of *T*_N_F_N_ for the 5-*m* series and *T*_N_F_I_ for the 6-*m* series show a similar dependence on *m* as described for *T*_NI_ for the 5-*m* series. The initial decrease in *T*_NI_ on increasing *m* is more pronounced, however, than that seen for the values of *T*_N_F_N_ or *T*_N_F_I_. It is noteworthy that although *T*_NI_ for 5-1 is 34 °C higher than that shown by 6-1, the values of *T*_N_F_N_ are in the opposite sense and is 7 °C higher for 6-1 than 5-1. This implies that other properties of the substituent other than size are important in determining *T*_N_F_N_. Indeed, across the complete series, *T*_N_F_I_ is higher for the 6-1 series than *T*_N_F_N_ for the corresponding member of the 5-*m* series. In terms of materials design, the lateral fluorine substituent has destabilised the N phase whereas it has stabilised the N_F_ phase to give a series of materials exhibiting the N_F_–I transition. This observation is consistent with the behaviour of structurally similar materials reported previously.^5^ The increase in the stability of the N_F_ phase associated with the addition of the fluorine atom may be attributed to changes in the molecular dipole moment and molecular polarizability. The average molecular dipole moment for members of the 5-*m* series is around 11.22 D and increases to about 12.20 D for the 6-*m* series. In itself, however, a large longitudinal dipole moment is not sufficient to drive the formation of the N_F_ phase. Fig. 10 compares the electrostatic potential surfaces for 5-2 and 6-2 and it is evident that the addition of the lateral fluorine atom has significantly changed the electron distribution in the local environment of the nitro group. In the framework of a molecular model developed to describe the N_F_ phase, the rod-like molecules are considered to possess longitudinal surface charge density waves and these interact inhibiting the formation of antiparallel structures.^37^ The parallel alignment of these rods is enhanced by reducing the amplitude of the charge density wave at either end of the molecule and this is presumably achieved by the addition of the lateral fluorine substituent that removes electron density from the nitro group.

**Fig. 11:**
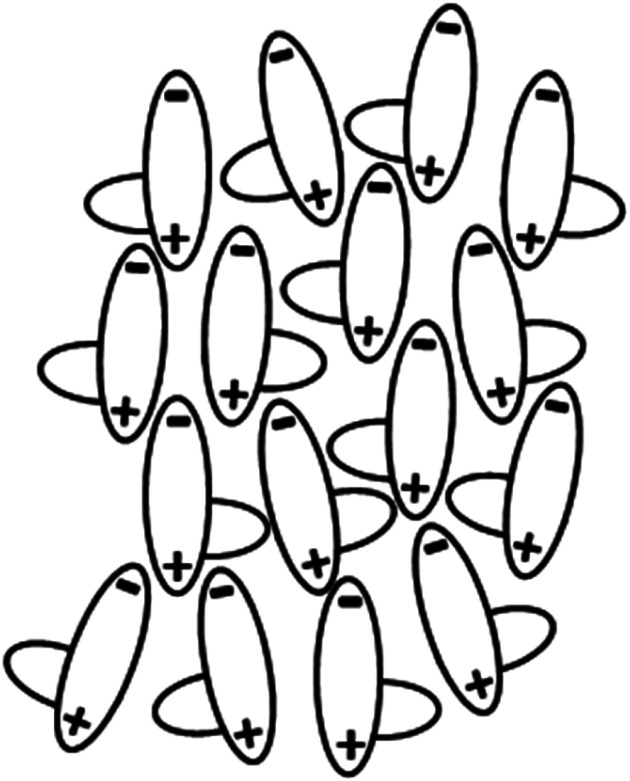
A sketch of the possible local molecular arrangement in the N_F_ phase containing molecules having a bulky lateral substituent.

As we have seen in both the 5-*m* and 6-*m* series, the N_F_ phase is observed for compounds containing long lateral alkyloxy chains. This is quite unlike the behaviour seen on increasing the length of a terminal chain which, even for short chains, results in the loss of N_F_ behaviour.^5^ This has been attributed to the formation of anti-parallel structures in order to minimise dipolar energy, and as chain length is increased molecular inhomogeneity drives smectic behaviour. By comparison, lateral chains suppress the formation of smectic phases by inhibiting lateral interactions between the mesogenic cores. This in turn may help to stabilise the formation of the N_F_ phase as shown schematically in Fig. 11.

## Conclusions

Our understanding of the structural features that promote the formation of the ferroelectric nematic phase is still at an early stage of development. Here we have seen that a bulky lateral alkyloxy chain stabilises the N_F_ phase relative to the N phase, and suggest that this may, at least in part, be due to the chain inhibiting lateral interactions between the mesogenic cores that otherwise may drive antiparallel dipolar associations. A comparison of the two series supports the view that the replacement of a hydrogen atom by a fluorine atom decreases *T*_NI_ but increases *T*_N_F_N_ and the 6-*m* series show direct N_F_–I transitions. The decrease in *T*_NI_ is associated with the change in structural anisotropy, whereas the increase in *T*_N_F_N_ is attributed to the change in electron distribution within the framework of a molecular model that describes the formation of the N_F_ phase in low molar mass materials.^37^ In terms of molecular design this ability of the N_F_ phase to tolerate bulky lateral substituents has been exploited, for example, in the design of chiral ferroelectric nematogens^29^ and other functionalities may now also be incorporated.

## Conflicts of interest

There are no conflicts to declare.

## Supplementary Material

RA-012-D2RA05628C-s001
