# Supplementary material for: The effect of a lateral alkyloxy chain on the ferroelectric nematic phase
Source: RSC Adv. 2022 Oct 14;12(45):29482–90. doi: 10.1039/d2ra05628c (PMC9562421; doi:10.1039/d2ra05628c)
Supplement: RA-012-D2RA05628C-s001 [file RA-012-D2RA05628C-s001.pdf]

Electronic Supplementary Material (ESI) for RSC Advances.
This journal is © The Royal Society of Chemistry 2022

The effect of a lateral alkyloxy chain on the ferroelectric nematic phase

Ewan Cruickshank, Rebecca Walker, John M.D. Storey, Corrie T. Imrie

Department of Chemistry, University of Aberdeen, Old Aberdeen, AB24 3UE, U.K.

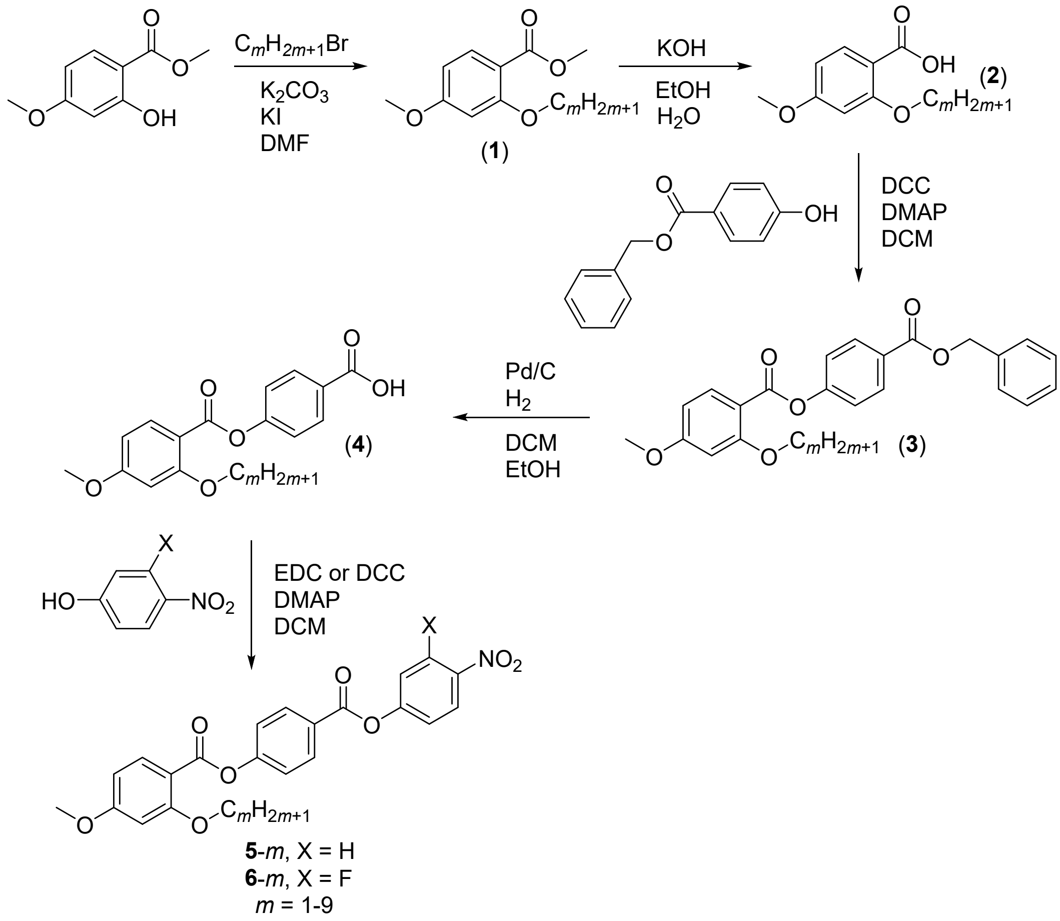

Synthesis and Analytical Data

Methyl 2-alkoxy-4-methoxybenzoates (1)

To a pre-dried flask flushed with argon and fitted with a condenser, methyl 4-methoxysilicylate (1 eq, 5.00 g, 0.0274 mol), potassium iodide (except *m* = 2) (1.1 eq, 5.00 g, 0.0301 mol), and potassium carbonate (2 eq, 7.57 g, 0.0548 mol) were combined in DMF (80 mL). To the mixture, the appropriate 1-bromoalkane (1.1 eq) was added (or in the case of *m* = 2, iodoethane (1 eq, 2.20 mL, 4.27 g, 0.0274 mol) and stirred at 90°C overnight. The quantities of the 1-bromoalkanes used in each reaction are listed in **Table 1**. The extent of the reaction was monitored by TLC using a suitable solvent system (RF values quoted in the product data). The reaction mixture was cooled to room temperature and poured into water (150 mL). The resulting suspension was extracted with ethyl acetate (2 x 250 mL). The organic

fractions were combined, washed with water (3 x 100 mL) and dried over anhydrous magnesium sulfate. The magnesium sulfate was removed using vacuum filtration and the solvent evaporated under vacuum to leave a yellow oil or solid. The product was carried forwards without any further purification.

Table 1. Quantities of 1-bromoalkanes used in the syntheses of the methyl 2-alkoxy-4-methoxybenzoates

m	1-Bromoalkane
3	2.73 mL, 3.70 g, 0.0301 mol
4	3.23 mL, 4.12 g, 0.0301 mol
5	3.73 mL, 4.55 g, 0.0301 mol
6	4.23 mL, 4.97 g, 0.0301 mol
7	4.73 mL, 5.39 g, 0.0301 mol
8	5.20 mL, 5.81 g, 0.0301 mol
9	5.75 mL, 6.24 g, 0.0301 mol

1.1 Methyl 2-ethoxy-4-methoxybenzoate

Yellow solid. Yield: 4.36 g, 75.6 %. RF: 0.559 (40 % ethyl acetate:60 % 40:60 petroleum ether). M.P = 47 °C

ν_{max}/cm^{-1}: 2982, 2941, 2842, 1726, 1688, 1669, 1603, 1575, 1505, 1440, 1423, 1393, 1370, 1326, 1256, 1206, 1192, 1132, 1111, 1090, 1029, 982, 970, 893, 847, 822, 770, 728, 695, 652, 625, 585, 540, 469

δ_H/ppm (400 MHz, $CDCl_3$): 7.83 (1 H, d, J 8.4 Hz, Ar-H), 6.47 (2 H, m, Ar-H), 4.08 (2 H, q, J 7.0 Hz, O-$\underline{CH_2}$-CH_3), 3.84 (3 H, s, (C=O)-O-$\underline{CH_3}$), 3.82 (3 H, s, O-$\underline{CH_3}$), 1.46 (3 H, t, J 7.0 Hz, O-CH_2-$\underline{CH_3}$)

δ_C/ppm (100 MHz, $CDCl_3$): 166.21, 164.09, 160.71, 133.73, 112.65, 104.65, 100.04, 64.60, 55.43, 51.58, 14.66

1.2 Methyl 4-methoxy-2-propoxybenzoate

Yellow oil. Yield: 5.19 g, 84.5 %. RF: 0.639 (40 % ethyl acetate:60 % 40:60 petroleum ether).

ν_{max}/cm^{-1}: 2848, 2878, 2840, 1724, 1697, 1607, 1575, 1505, 1436, 1390, 1325, 1297, 1248, 1203, 1170, 1138, 1085. 1038, 994, 965, 932, 833, 768, 698, 623, 600, 462

δ_H/ppm (400 MHz, $CDCl_3$): 7.80 (1 H, d, J 8.5 Hz, Ar-H), 6.43 (2 H, m, Ar-H), 3.93 (2 H, t, J 6.5 Hz, O-$\underline{CH_2}$-CH_2-), 3.82 (3 H, s, (C=O)-O-$\underline{CH_3}$), 3.79 (3 H, s, O-$\underline{CH_3}$), 1.84 (2 H, tq, J 6.5 Hz, 7.4 Hz, O-CH_2-$\underline{CH_2}$-CH_3), 1.05 (3 H, t, J 7.4 Hz, O-CH_2-CH_2-$\underline{CH_3}$)

δ_C/ppm (100 MHz, $CDCl_3$): 166.29, 164.09, 160.83, 133.73, 112.55, 104.59, 99.80, 70.32, 55.38, 51.51, 22.51, 10.52

1.3 Methyl 2-butoxy-4-methoxybenzoate

Yellow oil. Yield: 5.19 g, 79.5 %. RF: 0.611 (40 % ethyl acetate:60 % 40:60 petroleum ether).

ν_{max}/cm^{-1}: 2955, 2873, 2840, 1725, 1697, 1669, 1607, 1575, 1505, 1436, 1389, 1325, 1298, 1248, 1203, 1169, 1138, 1086, 1037, 1010, 965, 951, 833, 768, 698, 624, 600, 462

δ_H/ppm (400 MHz, $CDCl_3$): 7.81 (1 H, d, J 9.1 Hz, Ar-H), 6.44 (2 H, m, Ar-H), 3.98 (2 H, t, J 6.5 Hz, O-$\underline{CH_2}$-CH_2-), 3.82 (3 H, s, (C=O)-O-$\underline{CH_3}$), 3.79 (3 H, s, O-$\underline{CH_3}$), 1.79 (2 H, tt, J 6.5 Hz, 7.4 Hz,

$O-CH_2-\underline{CH_2}-CH_2-$), 1.50 (2 H, sext, J 7.4 Hz, $O-CH_2-CH_2-\underline{CH_2}-CH_3$), 0.96 (3 H, t, J 7.4 Hz, $O-CH_2-CH_2-CH_2-\underline{CH_3}$)

δ_C/ppm (100 MHz, $CDCl_3$): 166.28, 164.09, 160.84, 133.74, 112.56, 104.55, 99.80, 68.55, 55.38, 51.51, 31.16, 19.19, 13.81

1.4 Methyl 4-methoxy-2-pentoxybenzoate

Yellow solid. Yield: 5.40 g, 78.1 %. RF: 0.667 (40 % ethyl acetate:60 % 40:60 petroleum ether).

M.P = 46 °C

v_{max}/cm^{-1}: 2941, 2871, 1724, 1694, 1669, 1606, 1576, 1505, 1437, 1394, 1332, 1298, 1253, 1206, 1190, 1170, 1132, 1088, 1033, 1017, 967, 952, 837, 815, 773, 932, 695, 652, 625, 591, 460

δ_H/ppm (400 MHz, $CDCl_3$): 7.84 (1 H, d, J 8.5 Hz, Ar-H), 6.46 (2 H, m, Ar-H), 3.99 (2 H, t, J 6.6 Hz, $O-\underline{CH_2}-CH_2-$), 3.84 (3 H, s, (C=O)-$O-\underline{CH_3}$), 3.83 (3 H, s, $O-\underline{CH_3}$), 1.84 (2 H, tt, J 6.6 Hz, 7.2 Hz, $O-CH_2-\underline{CH_2}-CH_2-$), 1.43 (4 H, m, $O-CH_2-CH_2-\underline{CH_2}-\underline{CH_2}-CH_3$), 0.93 (3 H, t, J 7.2 Hz, $O-CH_2-CH_2-CH_2-CH_2-\underline{CH_3}$)

δ_C/ppm (100 MHz, $CDCl_3$): 166.39, 164.09, 160.83, 133.79, 112.63, 104.57, 99.87, 68.93, 55.44, 51.58, 28.80, 28.13, 22.41, 14.05

1.5 Methyl 2-hexyloxy-4-methoxybenzoate

Yellow oil. Yield: 5.51 g, 75.5 %. RF: 0.611 (40 % ethyl acetate:60 % 40:60 petroleum ether).

v_{max}/cm^{-1}: 2950, 2859, 1726, 1697, 1607, 1575, 1505, 1434, 1389, 1325, 1297, 1250, 1204, 1169, 1138, 1086, 1039, 964, 916, 835, 768, 699, 625, 600, 464

δ_H/ppm (400 MHz, $CDCl_3$): 7.84 (1 H, d, J 8.5 Hz, Ar-H), 6.47 (2 H, m, Ar-H), 4.00 (2 H, t, J 6.5 Hz, $O-\underline{CH_2}-CH_2-$), 3.85 (3 H, s, (C=O)-$O-\underline{CH_3}$), 3.83 (3 H, s, $O-\underline{CH_3}$), 1.83 (2 H, tt, J 6.5 Hz, 7.2 Hz, $O-CH_2-\underline{CH_2}-CH_2-$), 1.50 (2 H, m, $O-CH_2-CH_2-\underline{CH_2}-CH_2-$), 1.34 (4 H, m, $O-CH_2-CH_2-CH_2-\underline{CH_2}-\underline{CH_2}-CH_3$), 0.91 (3 H, t, J 7.0 Hz, $O-CH_2-CH_2-CH_2-CH_2-CH_2-\underline{CH_3}$)

δ_C/ppm (100 MHz, $CDCl_3$): 166.42, 164.09, 160.82, 133.80, 112.65, 104.57, 99.87, 68.95, 55.44, 51.59, 31.54, 29.08, 25.65, 22.61, 14.04

1.6 Methyl 2-heptyloxy-4-methoxybenzoate

Yellow oil. Yield: 6.44 g, 83.8 %. RF: 0.675 (40 % ethyl acetate:60 % 40:60 petroleum ether).

v_{max}/cm^{-1}: 2929, 2857, 1726, 1697, 1670, 1607, 175, 1505, 1441, 1389, 1325, 1297, 1249, 1203, 1169, 1138, 1086, 1037, 966, 833, 768, 725, 698, 625, 599, 461

δ_H/ppm (400 MHz, $CDCl_3$): 7.83 (1 H, d, J 8.5 Hz, Ar-H), 6.46 (2 H, m, Ar-H), 3.99 (2 H, t, J 6.6 Hz, $O-\underline{CH_2}-CH_2-$), 3.84 (3 H, s, (C=O)-$O-\underline{CH_3}$), 3.83 (3 H, s, $O-\underline{CH_3}$), 1.83 (2 H, tt, J 6.6 Hz, 7.0 Hz, $O-CH_2-\underline{CH_2}-CH_2-$), 1.48 (2 H, m, $O-CH_2-CH_2-\underline{CH_2}-CH_2-$), 1.34 (6 H, m, $O-CH_2-CH_2-CH_2-\underline{CH_2}-\underline{CH_2}-\underline{CH_2}-CH_3$), 0.89 (3 H, t, J 7.0 Hz, $O-CH_2-CH_2-CH_2-CH_2-CH_2-CH_2-\underline{CH_3}$)

δ_C/ppm (100 MHz, $CDCl_3$): 166.39, 164.09, 160.82, 133.79, 112.64, 104.56, 99.87, 68.94, 55.43, 51.57, 31.79, 29.12, 29.01, 25.92, 22.62, 14.09

1.7 Methyl 4-methoxy-2-octyloxybenzoate

Yellow oil. Yield: 7.33 g, 90.9 %. RF: 0.625 (40 % ethyl acetate:60 % 40:60 petroleum ether).

v_{max}/cm^{-1}: 2925, 2855, 1727, 1698, 1671, 1607, 1575, 1505, 1442, 1388, 1325, 1297, 1250, 1203, 1169, 1138, 1086, 1038, 966, 835, 769, 723, 698, 625, 599, 461

δ_H/ppm (400 MHz, $CDCl_3$): 7.82 (1 H, d, J 8.5 Hz, Ar-H), 6.45 (2 H, m, Ar-H), 3.98 (2 H, t, J 6.5 Hz, $O-\underline{CH_2}-CH_2-$), 3.83 (3 H, s, (C=O)-$O-\underline{CH_3}$), 3.81 (3 H, s, $O-\underline{CH_3}$), 1.82 (2 H, tt, J 6.5 Hz, 7.0 Hz,

O-CH_2-$\underline{CH_2}$-CH_2-), 1.48 (2 H, m, O-CH_2-CH_2-$\underline{CH_2}$-CH_2-), 1.29 (8 H, m, O-CH_2-CH_2-CH_2-$\underline{CH_2}$-$\underline{CH_2}$-$\underline{CH_2}$-$\underline{CH_2}$-CH_3), 0.87 (3 H, t, J 7.2 Hz, O-CH_2-CH_2-CH_2-CH_2-CH_2-CH_2-CH_2-$\underline{CH_3}$)
δ_C/ppm (100 MHz, $CDCl_3$): 166.37, 164.09, 163.77, 133.77, 112.60, 104.57, 99.84, 68.93, 55.41, 51.54, 31.82, 29.30, 29.24, 29.11, 25.95, 22.66, 14.09

1.8 Methyl 4-methoxy-2-nonyloxybenzoate

Yellow oil. Yield: 7.66 g, 90.6 %. RF: 0.625 (40 % ethyl acetate:60 % 40:60 petroleum ether).
ν_{max}/cm^{-1}: 2924, 2854, 1727, 1703, 1670, 1607, 1576, 1505, 1441, 1388, 1349, 1326, 1298, 1251, 1203, 1169, 1138, 1087, 1038, 952, 835, 769, 728, 698, 625, 599, 519, 461
δ_H/ppm (400 MHz, $CDCl_3$): 7.81 (1 H, d, J 8.4 Hz, Ar-H), 6.44 (2 H, m, Ar-H), 3.97 (2 H, t, J 6.5 Hz, O-$\underline{CH_2}$-CH_2-), 3.82 (3 H, s, (C=O)-O-$\underline{CH_3}$), 3.80 (3 H, s, O-$\underline{CH_3}$), 1.82 (2 H, tt, J 6.5 Hz, 7.1 Hz, O-CH_2-$\underline{CH_2}$-CH_2-), 1.47 (2 H, m, O-CH_2-CH_2-$\underline{CH_2}$-CH_2-), 1.27 (10 H, m, O-CH_2-CH_2-CH_2-$\underline{CH_2}$-$\underline{CH_2}$-$\underline{CH_2}$-$\underline{CH_2}$-$\underline{CH_2}$-CH_3), 0.85 (3 H, t, J 7.1 Hz, O-CH_2-CH_2-CH_2-CH_2-CH_2-CH_2-CH_2-CH_2-$\underline{CH_3}$)
δ_C/ppm (100 MHz, $CDCl_3$): 166.34, 164.08, 160.81, 133.76, 112.58, 104.56, 99.83, 68.91, 55.39, 51.52, 31.88, 29.54, 29.34, 29.26, 29.10, 25.94, 22.66, 14.09

2-Alkoxy-4-methoxybenzoic acids (2)

To a pre-dried flask flushed with argon and fitted with a condenser, potassium hydroxide (3 eq) was added to water. **Compound 1** (1 eq) was solubilised in EtOH, added to the flask and the resultant mixture stirred at reflux overnight. The quantities of the reagents used in each reaction are listed in **Table 2**. The extent of the reaction was monitored by TLC using an appropriate solvent system (RF values quoted in the product data). The reaction mixture was cooled to room temperature and the pH of the mixture was adjusted to 1 using 32% hydrochloric acid (25 mL). If a white solid precipitated after acidification it was collected by vacuum filtration. If no precipitate formed the reaction mixture was extracted with ethyl acetate (2 x 200 mL). The organic layers were combined, washed with water (2 x 100 mL) and dried over anhydrous magnesium sulfate. The magnesium sulfate was removed using vacuum filtration and the solvent evaporated under vacuum to leave a white or off-white solid. The product was carried forwards without any further purification (*m* = 5, 7, 8, 9 were recrystallised from hot ethanol (75 mL) to give a white solid).

Table 2. Quantities of reagents used in the syntheses of the 2-alkoxy-4-methoxybenzoic acids

m	(**1**)	Ethanol	Potassium Hydroxide	Water
2	5.00 g, 0.0238 mol	50 mL	4.00 g, 0.0714 mol	30 mL
3	5.00 g, 0.0223 mol	70 mL	3.75 g, 0.0669 mol	35 mL
4	5.00 g, 0.0210 mol	70 mL	3.53 g, 0.0630 mol	35 mL
5	5.20 g, 0.0206 mol	70 mL	3.47 g, 0.0618 mol	35 mL
6	3.78 g, 0.0142 mol	30 mL	2.39 g, 0.0426 mol	50 mL
7	6.00 g, 0.0214 mol	70 mL	3.60 g, 0.0642 mol	40 mL
8	6.50 g, 0.0221 mol	75 mL	3.72 g, 0.0663 mol	40 mL
9	7.00 g, 0.0227 mol	80 mL	3.82 g, 0.0681 mol	50 mL

2.1 2-Ethoxy-4-methoxybenzoic acid

White solid. Yield: 4.30 g, 92.0 %. RF: 0.306 (40 % ethyl acetate:60 % 40:60 petroleum ether). M.P = 121 °C

ν_{max}/cm^{-1}: 2985, 2874, 1666, 613, 1569, 1506, 1451, 1413, 1386, 1311, 1275, 1254, 1202, 1174, 1150, 1114, 1094, 1034, 918, 890, 831, 815, 793, 765, 736, 690, 632, 616, 578, 490, 464, 410

δ_H/ppm (400 MHz, DMSO-d_6): 12.07 (1 H, s, OH), 7.68 (1 H, d, J 8.6 Hz, Ar-H), 6.59 (1 H, d, J 2.2 Hz, Ar-H), 6.56 (1 H, dd, J 8.6 Hz, 2.2 Hz, Ar-H), 4.08 (2 H, q, J 7.0 Hz, O-$\underline{CH_2}$-CH_3), 3.80 (3 H, s, O-$\underline{CH_3}$), 1.32 (3 H, t, J 7.0 Hz, O-CH_2-$\underline{CH_3}$)

δ_C/ppm (100 MHz, DMSO-d_6): 166.97, 163.96, 160.22, 133.60, 113.52, 105.72, 100.24, 64.58, 55.92, 14.97

2.2 4-Methoxy-2-propoxybenzoic acid

White solid. Yield: 3.74 g, 79.8 %. RF: 0.541 (100 % ethyl acetate). M.P = 88 °C

ν_{max}/cm^{-1}: 2972, 2881, 1679, 1660, 1601, 1570, 1507, 1462, 1446, 1409, 1393, 1333, 1309, 1242, 1213, 1180, 1146, 1091, 1062, 1030, 1019, 993, 926, 834, 817, 793, 779, 698, 637, 594, 570, 490, 456, 417

δ_H/ppm (400 MHz, DMSO-d_6): 12.08 (1 H, s, OH), 7.68 (1 H, d, J 8.6 Hz, Ar-H), 6.58 (1 H, d, J 2.3 Hz, Ar-H), 6.55 (1 H, dd, J 8.6 Hz, 2.3 Hz, Ar-H), 3.98 (2 H, t, J 6.4 Hz, O-$\underline{CH_2}$-CH_2-), 3.80 (3 H, s, O-$\underline{CH_3}$), 1.72 (2 H, tq, J 6.4 Hz, 7.3 Hz, O-CH_2-$\underline{CH_2}$-CH_3), 0.99 (3 H, t, J 7.3 Hz, O-CH_2-CH_2-$\underline{CH_3}$)

δ_C/ppm (100 MHz, DMSO-d_6): 167.03, 163.97, 160.42, 133.59, 113.44, 105.68, 100.06, 70.19, 55.92, 22.46, 10.92

2.3 2-Butoxy-4-methoxybenzoic acid

White solid. Yield: 3.33 g, 70.7 %. RF: 0.568 (100 % ethyl acetate). M.P = 83 °C

ν_{max}/cm^{-1}: 2942, 2872, 1681, 1657, 1600, 1570, 1506, 1446, 1410, 1393, 1331, 1305, 1243, 1212, 1178, 1146, 1091, 1064, 1029, 1011, 988, 959, 938, 835, 812, 795, 777, 739, 694, 652, 640, 594, 571, 524, 474, 458, 418

δ_H/ppm (400 MHz, DMSO-d_6): 12.07 (1 H, s, OH), 7.68 (1 H, d, J 8.6 Hz, Ar-H), 6.59 (1 H, d, J 2.3 Hz, Ar-H), 6.55 (1 H, dd, J 8.6 Hz, 2.3 Hz, Ar-H), 4.02 (2 H, t, J 6.3 Hz, O-$\underline{CH_2}$-CH_2-), 3.80 (3 H, s, O-$\underline{CH_3}$), 1.69 (2 H, tt, J 6.3 Hz, 7.4 Hz, O-CH_2-$\underline{CH_2}$-CH_2-), 1.46 (2 H, sext, J 7.4 Hz, O-CH_2-CH_2-$\underline{CH_2}$-CH_3), 0.92 (3 H, t, J 7.4 Hz, O-CH_2-CH_2-CH_2-$\underline{CH_3}$)

δ_C/ppm (100 MHz, DMSO-d_6): 167.02, 163.96, 160.41, 133.56, 113.47, 105.68, 100.07, 68.42, 55.93, 31.12, 19.12, 14.14

2.4 4-Methoxy-2-pentoxybenzoic acid

White solid. Yield: 2.21 g, 45.0 %. RF: 0.568 (100 % ethyl acetate). M.P = 75 °C

ν_{max}/cm^{-1}: 2942, 2866, 2547, 1686, 1659, 1605, 1569, 1505, 1450, 1430, 1405, 1391, 1307, 1271, 1252, 1206, 1177, 1150, 1125, 1110, 1094, 1039, 1021, 940, 836, 824, 792, 771, 736, 694, 620, 584 ,499, 460, 432

δ_H/ppm (400 MHz, DMSO-d_6): 12.06 (1 H, s, OH), 7.68 (1 H, d, J 8.6 Hz, Ar-H), 6.59 (1 H, d, J 2.3 Hz, Ar-H), 6.55 (1 H, dd, J 8.6 Hz, 2.3 Hz, Ar-H), 4.01 (2 H, t, J 6.4 Hz, O-$\underline{CH_2}$-CH_2-), 3.80 (3 H, s, O-$\underline{CH_3}$), 1.71 (2 H, tt, J 6.4 Hz, 6.8 Hz, O-CH_2-$\underline{CH_2}$-CH_2-), 1.37 (4 H, m, O-CH_2-CH_2-$\underline{CH_2}$-$\underline{CH_2}$-CH_3), 0.89 (3 H, t, J 7.2 Hz, O-CH_2-CH_2-CH_2-CH_2-$\underline{CH_3}$)

δ_C/ppm (100 MHz, DMSO-d_6): 167.02, 163.96, 160.40, 133.55, 113.48, 105.69, 100.09, 68.73, 55.93, 28.71, 28.07, 22.27, 14.40

2.5 2-Hexyloxy-4-methoxybenzoic acid

Off-white solid. Yield: 3.23 g, 90.1 %. RF: 0.378 (40 % ethyl acetate:60 % 40:60 petroleum ether). M.P = 57 °C

v_{max}/cm^{-1}: 2948, 2867, 1680, 1663, 1601, 1571, 1507, 1449, 1449, 1411, 1391, 1331, 1243, 1210, 1176, 1147, 1092, 979, 948, 834, 814, 795, 777, 728, 694, 654, 642, 626, 595, 570, 523, 489, 461, 437

δ_H/ppm (400 MHz, DMSO-d_6): 11.51 (1 H, s, OH), 7.68 (1 H, d, J 8.6 Hz, Ar-H), 6.59 (1 H, d, J 2.3 Hz, Ar-H), 6.55 (1 H, dd, J 8.6 Hz, 2.3 Hz, Ar-H), 4.01 (2 H, t, J 6.4 Hz, O-$\underline{CH_2}$-CH_2-), 3.80 (3 H, s, O-$\underline{CH_3}$), 1.70 (2 H, tt, J 6.4 Hz, 6.8 Hz, O-CH_2-$\underline{CH_2}$-CH_2-), 1.44 (2 H, m, O-CH_2-CH_2-$\underline{CH_2}$-CH_2-), 1.30 (4 H, m, O-CH_2-CH_2-CH_2-$\underline{CH_2}$-$\underline{CH_2}$-CH_3), 0.87 (3 H, t, J 7.1 Hz, O-CH_2-CH_2-CH_2-CH_2-CH_2-$\underline{CH_3}$)

δ_C/ppm (100 MHz, DMSO-d_6): 167.00, 163.96, 160.42, 133.57, 113.45, 105.67, 100.06, 68.73, 55.91, 31.37, 28.98, 25.51, 22.53, 14.34

2.6 2-Heptyloxy-4-methoxybenzoic acid

White solid. Yield: 2.94 g, 51.6 %. RF: 0.410 (40 % ethyl acetate:60 % 40:60 petroleum ether). M.P = 60 °C

v_{max}/cm^{-1}: 2938, 2853, 1659, 1638, 1604, 1568, 1505, 1447, 1410, 1392, 1382, 1310, 1273, 1247, 1202, 1172, 1148, 1110, 1094, 1064, 1034, 1006, 929, 861, 828, 796, 770, 724, 693, 648, 633, 615, 584, 526, 465, 407

δ_H/ppm (400 MHz, DMSO-d_6): 12.05 (1 H, s, OH), 7.68 (1 H, d, J 8.6 Hz, Ar-H), 6.59 (1 H, d, J 2.3 Hz, Ar-H), 6.55 (1 H, dd, J 8.6 Hz, 2.3 Hz, Ar-H), 4.01 (2 H, t, J 6.4 Hz, O-$\underline{CH_2}$-CH_2-), 3.80 (3 H, s, O-$\underline{CH_3}$), 1.70 (2 H, tt, J 6.4 Hz, 6.8 Hz, O-CH_2-$\underline{CH_2}$-CH_2-), 1.44 (2 H, tt, 6.8 Hz, 7.0 Hz, O-CH_2-CH_2-$\underline{CH_2}$-CH_2-), 1.28 (6 H, m, O-CH_2-CH_2-CH_2-$\underline{CH_2}$-$\underline{CH_2}$-$\underline{CH_2}$-CH_3), 0.86 (3 H, t, J 7.0 Hz, O-CH_2-CH_2-CH_2-CH_2-CH_2-CH_2-$\underline{CH_3}$)

δ_C/ppm (100 MHz, DMSO-d_6): 166.99, 163.96, 160.42, 133.57, 113.45, 105.67, 100.07, 68.72, 55.91, 31.72, 29.03, 28.83, 25.81, 22.49, 14.42

2.7 4-Methoxy-2-octyloxybenzoic acid

White solid. Yield: 2.81 g, 46.8 %. RF: 0.410 (40 % ethyl acetate:60 % 40:60 petroleum ether). M.P = 56 °C

v_{max}/cm^{-1}: 2922, 2850, 1660, 1605, 1568, 1505, 1446, 1410, 1386, 1310, 1272, 1250, 1202, 1173, 1148, 1111, 1094, 1067, 1037, 1016, 939, 828, 791, 771, 726, 694, 634, 646, 585, 525, 464, 419

δ_H/ppm (400 MHz, DMSO-d_6): 12.05 (1 H, s, OH), 7.68 (1 H, d, J 8.6 Hz, Ar-H), 6.59 (1 H, d, J 2.2 Hz, Ar-H), 6.55 (1 H, dd, J 8.6 Hz, 2.2 Hz, Ar-H), 4.01 (2 H, t, J 6.4 Hz, O-$\underline{CH_2}$-CH_2-), 3.80 (3 H, s, O-$\underline{CH_3}$), 1.71 (2 H, tt, J 6.4 Hz, 7.0 Hz, O-CH_2-$\underline{CH_2}$-CH_2-), 1.44 (2 H, quin, 7.0 Hz, O-CH_2-CH_2-$\underline{CH_2}$-CH_2-), 1.29 (8 H, m, O-CH_2-CH_2-CH_2-$\underline{CH_2}$-$\underline{CH_2}$-$\underline{CH_2}$-$\underline{CH_2}$-CH_3), 0.86 (3 H, t, J 7.0 Hz, O-CH_2-CH_2-CH_2-CH_2-CH_2-CH_2-CH_2-$\underline{CH_3}$)

δ_C/ppm (100 MHz, DMSO-d_6): 166.98, 163.96, 160.42, 133.57, 113.45, 105.67, 100.08, 68.73, 55.91, 31.69, 29.15, 29.13, 29.03, 25.86, 22.56, 14.42

2.8 4-Methoxy-2-nonyloxybenzoic acid

White solid. Yield: 3.55 g, 53.1 %. RF: 0.410 (40 % ethyl acetate:60 % 40:60 petroleum ether). M.P = 56 °C

v_{max}/cm^{-1}: = 2923, 2848, 1658, 1604, 1567, 1508, 1464, 1447, 1410, 1388, 1309, 1271, 1251, 1201, 1174, 1148, 1111, 1094, 1069, 1038, 1014, 977, 926, 906, 827, 795, 771, 724, 693, 634, 616, 584, 528, 482, 460, 427

δ_H/ppm (400 MHz, DMSO-d_6): 12.04 (1 H, s, OH), 7.68 (1 H, d, J 8.7 Hz, Ar-H), 6.58 (1 H, d, J 2.2 Hz, Ar-H), 6.54 (1 H, dd, J 8.7 Hz, 2.2 Hz, Ar-H), 4.00 (2 H, t, J 6.4 Hz, O-$\underline{CH_2}$-CH_2-), 3.80 (3 H, s, O-$\underline{CH_3}$), 1.70 (2 H, tt, J 6.4 Hz, 6.9 Hz, O-CH_2-$\underline{CH_2}$-CH_2-), 1.43 (2 H, quin, 6.9 Hz, O-CH_2-CH_2-$\underline{CH_2}$-CH_2-), 1.25 (10 H, m, O-CH_2-CH_2-CH_2-$\underline{CH_2}$-$\underline{CH_2}$-$\underline{CH_2}$-$\underline{CH_2}$-$\underline{CH_2}$-CH_3), 0.85 (3 H, t, J 6.9 Hz, O-CH_2-CH_2-CH_2-CH_2-CH_2-CH_2-CH_2-CH_2-$\underline{CH_3}$)
δ_C/ppm (100 MHz, DMSO-d_6): 166.97, 163.97, 160.42, 133.59, 113.44, 105.65, 100.06, 68.72, 55.89, 31.77, 29.47, 29.19, 29.12, 29.04, 25.86, 22.57, 14.40

4-[(Benzyloxy)carbonyl]phenyl 2-alkoxy-4-methoxybenzoates (3)

To a pre-dried flask flushed with argon, **Compound 2** (1 eq), benzyl 4-hydroxybenzoate (1.1 eq) and 4-dimethylaminopyridine (0.13 eq) were added. The solids were solubilised with dichloromethane (80 mL) and stirred for 10 min before *N,N'*-dicyclohexylcarbodiimide (1.3 eq) was added to the flask and the reaction was allowed to proceed overnight. The quantities of the reagents used in each reaction are listed in **Table 3**. The extent of the reaction was monitored by TLC using an appropriate solvent system (RF values quoted in the product data). The white precipitate which formed was removed by vacuum filtration and the filtrate collected. The collected solvent was evaporated under vacuum to leave a white solid which was recrystallised from hot ethanol (100 mL).

Table 3. Quantities of reagents used in the syntheses of the 4-[(benzyloxy)carbonyl]phenyl 2-alkoxy-4-methoxybenzoates

m	(**2**)	Benzyl 4-Hydroxybenzoate	4-Dimethylaminopyridine	*N,N'*-Dicyclohexylcarbodiimide
2	2.50 g, 0.0127 mol	3.20 g, 0.0140 mol	0.202 g, 1.65×10^{-3} mol	3.40 g, 0.0165 mol
3	1.80 g, 8.56×10^{-3} mol	2.15 g, 9.42×10^{-3} mol	0.136 g, 1.11×10^{-3} mol	2.29 g, 0.0111 mol
4	3.00 g, 0.0134 mol	3.36 g, 0.0147 mol	0.213 g, 1.74×10^{-3} mol	3.59 g, 0.0174 mol
5	2.00 g, 8.39×10^{-3} mol	2.11 g, 9.23×10^{-3} mol	0.133 g, 1.09×10^{-3} mol	2.25 g, 0.0109 mol
6	1.01 g, 4.00×10^{-3} mol	1.00 g, 4.40×10^{-3} mol	0.0635 g, 5.20×10^{-4} mol	1.07 g, 5.20×10^{-3} mol
7	2.70 g, 0.0101 mol	2.53 g, 0.0111 mol	0.160 g, 1.31×10^{-3} mol	2.70 g, 0.0131 mol
8	2.70 g, 9.63×10^{-3} mol	2.42 g, 0.0106 mol	0.152 g, 1.25×10^{-3} mol	2.58 g, 0.0125 mol
9	3.30 g, 0.0112 mol	2.81 g, 0.0123 mol	0.178 g, 1.46×10^{-3} mol	3.01 g, 0.0146 mol

3.1 4-[(Benzyloxy)carbonyl]phenyl 2-ethoxy-4-methoxybenzoate

White solid. Yield: 4.47 g, 86.6 %. RF: 0.590 (40 % ethyl acetate:60 % 40:60 petroleum ether). M.P = 95 °C

ν_{max}/cm^{-1}: 2992, 2973, 2937, 1742, 1717, 1603, 1569, 1500, 1453, 1441, 1427, 1412, 1397, 1299, 1274, 1241, 1199, 1173, 1164 ,1142, 1122, 1107, 1096, 1061, 1035, 1010, 960, 877, 827, 811, 789, 765, 757, 740, 699, 689, 663, 614, 597, 583 ,539, 526, 508, 460
δ_H/ppm (400 MHz, DMSO-d_6): 8.07 (2 H, d, J 8.5 Hz, Ar-H), 7.94 (1 H, d, J 8.7 Hz, Ar-H), 7.48 (2 H, m, Ar-H), 7.39 (5 H, m, Ar-H), 6.69 (1 H, d, J 2.2 Hz, Ar-H), 6.56 (1 H, dd, J 8.7 Hz, 2.2 Hz, Ar-H), 5.37 (2 H, s, (C=O)-O-$\underline{CH_2}$-Ar), 4.14 (2 H, q, J 6.9 Hz, O-$\underline{CH_2}$-CH_3), 3.86 (3 H, s, O-$\underline{CH_3}$), 1.33 (3 H, t, J 6.9 Hz, O-CH_2-$\underline{CH_3}$)
δ_C/ppm (100 MHz, DMSO-d_6): 165.42, 165.28, 163.09, 161.48, 155.23, 136.57, 134.37, 131.33, 129.00, 128.59, 128.43, 127.31, 122.95, 110.58, 106.25, 100.28, 66.73, 64.73, 56.16, 14.91

3.2 4-[(Benzyloxy)carbonyl]phenyl 4-methoxy-2-propoxybenzoate
White solid. Yield: 2.78 g, 77.2 %. RF: 0.611 (40 % ethyl acetate:60 % 40:60 petroleum ether). M.P = 82 °C
ν_{max}/cm^{-1}: 2996, 2969, 1738, 1707, 1600, 1569, 1497, 1457, 1442, 1429, 1392, 1375, 1299, 1268, 1243, 1195, 1170, 1163, 1141, 1110, 1094, 1049, 1007, 980, 936, 914, 891, 866, 820, 812, 755, 693, 658, 614, 595, 543, 510, 464
δ_H/ppm (400 MHz, $CDCl_3$): 8.14 (2 H, d, J 8.9 Hz, Ar-H), 8.04 (1 H, d, J 8.8 Hz, Ar-H), 7.40 (5 H, m, Ar-H), 7.28 (2 H, d, J 8.9 Hz, Ar-H), 6.55 (1 H, dd, J 8.8 Hz, 2.3 Hz, Ar-H), 6.51 (1 H, d, J 2.3 Hz, Ar-H), 5.37 (2 H, s, (C=O)-O-$\underline{CH_2}$-Ar), 4.01 (2 H, t, J 6.4 Hz, O-$\underline{CH_2}$-CH_2-), 3.88 (3 H, s, O-$\underline{CH_3}$), 1.87 (2 H, tq, J 6.4 Hz, 7.4 Hz, O-CH_2-$\underline{CH_2}$-CH_3), 1.06 (3 H, t, J 7.4 Hz, O-CH_2-CH_2-$\underline{CH_3}$)
δ_C/ppm (100 MHz, $CDCl_3$): 165.82, 165.07, 163.38, 161.81, 155.11, 136.05, 134.57, 131.25, 128.61, 128.25, 128.14, 127.26, 122.00, 110.97, 104.91, 99.73, 70.43, 66.73, 55.57, 22.52, 10.63

3.3 4-[(Benzyloxy)carbonyl]phenyl 2-butoxy-4-methoxybenzoate
White solid. Yield: 4.45 g, 76.4 %. RF: 0.583 (40 % ethyl acetate:60 % 40:60 petroleum ether). M.P = 84 °C
ν_{max}/cm^{-1}: 2963, 2935, 2870, 1733, 1716, 1603, 1568, 1502, 1455, 1430, 1409, 1394, 1373, 1303, 1268, 1248, 1194, 1157, 1139, 1112, 1092, 1049, 1034, 1005, 912, 886, 848, 815, 754, 691, 659, 618, 595, 553, 527, 505, 467
δ_H/ppm (400 MHz, $CDCl_3$): 8.14 (2 H, d, J 8.6 Hz, Ar-H), 8.04 (1 H, d, J 8.7 Hz, Ar-H), 7.41 (5 H, m, Ar-H), 7.28 (2 H, d, J 8.6 Hz, Ar-H), 6.55 (1 H, dd, J 8.7 Hz, 2.3 Hz, Ar-H), 6.51 (1 H, d, J 2.3 Hz, Ar-H), 5.38 (2 H, s, (C=O)-O-$\underline{CH_2}$-Ar), 4.05 (2 H, t, J 6.4 Hz, O-$\underline{CH_2}$-CH_2-), 3.88 (3 H, s, O-$\underline{CH_3}$), 1.82 (2 H, tt, J 6.4 Hz, 6.9 Hz, O-CH_2-$\underline{CH_2}$-CH_2-), 1.52 (2 H, tq, J 6.9 Hz, 7.4 Hz, O-CH_2-CH_2-$\underline{CH_2}$-CH_3), 0.94 (3 H, t, J 7.4 Hz, O-CH_2-CH_2-CH_2-$\underline{CH_3}$)
δ_C/ppm (100 MHz, $CDCl_3$): 165.82, 165.07, 163.35, 161.82, 155.11, 136.05, 134.57, 131.25, 128.61, 128.25, 128.15, 127.25, 121.99, 110.97, 104.88, 99.72, 68.61, 66.73, 55.57, 31.15, 19.21, 13.79

3.4 4-[(Benzyloxy)carbonyl]phenyl 4-methoxy-2-pentoxybenzoate
White solid. Yield: 3.03 g, 80.5 %. RF: 0.583 (40 % ethyl acetate:60 % 40:60 petroleum ether). M.P = 85 °C
ν_{max}/cm^{-1}: 2952, 2870, 1740, 1604, 1569, 1504, 1466, 1452, 1431, 1393, 1376, 1300, 1271, 1238, 1198, 1175, 1164, 1144, 1106, 1097, 1045, 1009, 934, 912, 886, 641, 757, 701, 692, 623, 593, 561, 519, 507
δ_H/ppm (400 MHz, $CDCl_3$): 8.14 (2 H, d, J 8.6 Hz, Ar-H), 8.04 (1 H, d, J 8.8 Hz, Ar-H), 7.41 (5 H, m, Ar-H), 7.28 (2 H, d, J 8.6 Hz, Ar-H), 6.55 (1 H, dd, J 8.8 Hz, 2.3 Hz, Ar-H), 6.51 (1 H, d, J 2.3 Hz,

Ar-H), 5.38 (2 H, s, (C=O)-O-$\underline{CH_2}$-Ar), 4.04 (2 H, t, J 6.5 Hz, O-$\underline{CH_2}$-CH_2-), 3.88 (3 H, s, O-$\underline{CH_3}$), 1.84 (2 H, tt, J 6.5 Hz, 7.0 Hz, O-CH_2-$\underline{CH_2}$-CH_2-), 1.47 (2 H, m, O-CH_2-CH_2-$\underline{CH_2}$-CH_2-) 1.35 (2 H, m, O-CH_2-CH_2-CH_2-$\underline{CH_2}$-CH_3), 0.88 (3 H, t, J 7.2 Hz, O-CH_2-CH_2-CH_2-CH_2-$\underline{CH_3}$)
δ_C/ppm (100 MHz, $CDCl_3$): 165.83, 165.06, 163.43, 161.78, 155.12, 136.05, 134.58, 131.24, 128.62, 128.25, 128.15, 127.25, 121.99, 111.00, 104.89, 99.73, 68.96, 66.74, 55.57, 28.81, 28.14, 22.39, 13.99

3.5 4-[(Benzyloxy)carbonyl]phenyl 2-hexyloxy-4-methoxybenzoate
Off-white solid. Yield: 1.37 g, 73.8 %. RF: 0.540 (40 % ethyl acetate:60 % 40:60 petroleum ether). M.P = 69 °C
v_{max}/cm^{-1}: 2946, 2921, 2856, 1740, 1703, 1603, 1569, 1503, 1449, 1430, 1413, 1386, 1376, 1320, 1301, 1266, 1243, 1195, 1174, 1162, 1144, 1107, 1093, 1081, 1041, 1020, 1010, 996, 961, 939, 916, 889, 835, 798, 756, 697, 660, 618, 598, 543, 518
δ_H/ppm (400 MHz, $CDCl_3$): 8.14 (2 H, d, J 8.5 Hz, Ar-H), 8.04 (1 H, d, J 8.7 Hz, Ar-H), 7.40 (5 H, m, Ar-H), 7.28 (2 H, d, J 8.5 Hz, Ar-H), 6.55 (1 H, dd, J 8.7 Hz, 2.3 Hz, Ar-H), 6.51 (1 H, d, J 2.3 Hz, Ar-H), 5.38 (2 H, s, (C=O)-O-$\underline{CH_2}$-Ar), 4.04 (2 H, t, J 6.5 Hz, O-$\underline{CH_2}$-CH_2-), 3.88 (3 H, s, O-$\underline{CH_3}$), 1.84 (2 H, tt, J 6.4 Hz, 6.9 Hz, O-CH_2-$\underline{CH_2}$-CH_2-), 1.49 (2 H, tt, J 6.9 Hz, 7.1 Hz, O-CH_2-CH_2-$\underline{CH_2}$-CH_2-), 1.29 (4 H, m, O-CH_2-CH_2-CH_2-$\underline{CH_2}$-$\underline{CH_2}$-CH_3), 0.85 (3 H, t, J 7.1 Hz, O-CH_2-CH_2-CH_2-CH_2-CH_2-$\underline{CH_3}$)
δ_C/ppm (100 MHz, $CDCl_3$): 165.83, 165.06, 163.44, 161.78, 155.12, 136.05, 134.58, 131.23, 128.62, 128.26, 128.14, 127.24, 121.99, 111.00, 104.89, 99.73, 68.97, 66.74, 55.57, 31.51, 29.09, 25.68, 22.56, 14.02

3.6 4-[(Benzyloxy)carbonyl]phenyl 2-heptyloxy-4-methoxybenzoate
White solid. Yield: 2.81 g, 58.5 %. RF: 0.575 (40 % ethyl acetate:60 % 40:60 petroleum ether). M.P = 71 °C
v_{max}/cm^{-1}: 2923, 2850, 1744, 1703, 1602, 1570, 1505, 1467, 1444, 1430, 1413, 1377, 1302, 1268, 1244, 1202, 1174, 1165, 1148, 1109, 1096, 1048, 1022, 1010, 966, 889, 832, 790, 759, 734, 695, 661, 617, 601, 545, 521, 504, 458
δ_H/ppm (400 MHz, $CDCl_3$): 8.14 (2 H, d, J 8.7 Hz, Ar-H), 8.03 (1 H, d, J 8.8 Hz, Ar-H), 7.42 (5 H, m, Ar-H), 7.28 (2 H, d, J 8.7 Hz, Ar-H), 6.55 (1 H, dd, J 8.8 Hz, 2.3 Hz, Ar-H), 6.51 (1 H, d, J 2.3 Hz, Ar-H), 5.37 (2 H, s, (C=O)-O-$\underline{CH_2}$-Ar), 4.04 (2 H, t, J 6.5 Hz, O-$\underline{CH_2}$-CH_2-), 3.88 (3 H, s, O-$\underline{CH_3}$), 1.83 (2 H, tt, J 6.5 Hz, 7.0 Hz, O-CH_2-$\underline{CH_2}$-CH_2-), 1.47 (2 H, quin, J 7.0 Hz, O-CH_2-CH_2-$\underline{CH_2}$-CH_2-), 1.27 (6 H, m, O-CH_2-CH_2-CH_2-$\underline{CH_2}$-$\underline{CH_2}$-$\underline{CH_2}$-CH_3), 0.85 (3 H, t, J 7.0 Hz, O-CH_2-CH_2-CH_2-CH_2-CH_2-CH_2-$\underline{CH_3}$)
δ_C/ppm (100 MHz, $CDCl_3$): 165.82, 165.05, 163.45, 161.77, 155.12, 136.05, 134.57, 131.23, 128.61, 128.25, 128.14, 127.24, 121.99, 111.01, 104.88, 99.74, 68.97, 66.73, 55.57, 31.73, 29.13, 29.00, 25.95, 22.59, 14.07

3.7 4-[(Benzyloxy)carbonyl]phenyl 4-methoxy-2-octyloxybenzoate
White solid. Yield: 3.92 g, 83.0 %. RF: 0.550 (40 % ethyl acetate:60 % 40:60 petroleum ether). M.P = 86 °C
v_{max}/cm^{-1}: 2919, 2851, 1742, 1707, 1602, 1571, 1505, 1470, 1456, 1445, 1430, 1413, 1389, 1376, 1302, 1267, 1244, 1203, 1176, 1165, 1148, 1110, 1096, 1049, 1022, 1013, 974, 908, 888, 790, 764, 758, 734, 695, 660, 648, 617, 603, 545, 523, 505, 455
δ_H/ppm (400 MHz, $CDCl_3$): 8.13 (2 H, d, J 8.8 Hz, Ar-H), 8.03 (1 H, d, J 8.7 Hz, Ar-H), 7.41 (5 H, m, Ar-H), 7.28 (2 H, d, J 8.8 Hz, Ar-H), 6.55 (1 H, dd, J 8.7 Hz, 2.3 Hz, Ar-H), 6.51 (1 H, d, J 2.3 Hz,

Ar-H), 5.37 (2 H, s, (C=O)-O-$\underline{CH_2}$-Ar), 4.04 (2 H, t, J 6.5 Hz, O-$\underline{CH_2}$-CH_2-), 3.88 (3 H, s, O-$\underline{CH_3}$), 1.83 (2 H, tt, J 6.5 Hz, 7.1 Hz, O-CH_2-$\underline{CH_2}$-CH_2-), 1.47 (2 H, quin, J 7.1 Hz, O-CH_2-CH_2-$\underline{CH_2}$-CH_2-), 1.26 (8 H, m, O-CH_2-CH_2-CH_2-$\underline{CH_2}$-$\underline{CH_2}$-$\underline{CH_2}$-$\underline{CH_2}$-CH_3), 0.85 (3 H, t, J 7.1 Hz, O-CH_2-CH_2-CH_2-CH_2-CH_2-CH_2-CH_2-$\underline{CH_3}$)
δ_C/ppm (100 MHz, $CDCl_3$): 165.82, 165.05, 163.46, 161.77, 155.11, 136.05, 134.57, 131.23, 128.61, 128.25, 128.14, 127.24, 121.98, 111.01, 104.88, 99.74, 68.97, 66.73, 55.57, 31.79, 29.30, 29.20, 29.12, 26.00, 22.64, 14.09

3.8 4-[(Benzyloxy)carbonyl]phenyl 4-methoxy-2-nonyloxybenzoate
White solid. Yield: 3.85 g, 68.1 %. RF: 0.650 (40 % ethyl acetate:60 % 40:60 petroleum ether). M.P = 65 °C
ν_{max}/cm^{-1}: = 2922, 2851, 1742, 1706, 1602, 1569, 1504, 1456, 1430, 1413, 1376, 1301, 1267, 1243, 1200, 1175, 1162, 1146, 1106, 1093, 1051, 1022, 1012, 981, 912, 890, 833, 791, 761, 750, 696, 660, 616, 600, 548, 508, 468
δ_H/ppm (400 MHz, $CDCl_3$): 8.13 (2 H, d, J 8.8 Hz, Ar-H), 8.03 (1 H, d, J 8.8 Hz, Ar-H), 7.41 (5 H, m, Ar-H), 7.29 (2 H, d, J 8.8 Hz, Ar-H), 6.53 (1 H, dd, J 8.8 Hz, 2.3 Hz, Ar-H), 6.51 (1 H, d, J 2.3 Hz, Ar-H), 5.37 (2 H, s, (C=O)-O-$\underline{CH_2}$-Ar), 4.04 (2 H, t, J 6.5 Hz, O-$\underline{CH_2}$-CH_2-), 3.88 (3 H, s, O-$\underline{CH_3}$), 1.83 (2 H, tt, J 6.5 Hz, 7.0 Hz, O-CH_2-$\underline{CH_2}$-CH_2-), 1.47 (2 H, quin, J 7.0 Hz, O-CH_2-CH_2-$\underline{CH_2}$-CH_2-), 1.25 (10 H, m, O-CH_2-CH_2-CH_2-$\underline{CH_2}$-$\underline{CH_2}$-$\underline{CH_2}$-$\underline{CH_2}$-$\underline{CH_2}$-CH_3), 0.86 (3 H, t, J 7.0 Hz, O-CH_2-CH_2-CH_2-CH_2-CH_2-CH_2-CH_2-CH_2-$\underline{CH_3}$)
δ_C/ppm (100 MHz, $CDCl_3$): 165.82, 165.05, 163.45, 161.78, 155.11, 136.05, 134.58, 131.23, 128.61, 128.25, 128.14, 127.24, 121.99, 111.01, 104.88, 99.73, 68.97, 66.73, 55.57, 31.86, 29.49, 29.33, 29.23, 29.12, 25.99, 22.65, 14.10

4-(2-Alkoxy-4-methoxybenzoyloxy)benzoic acids (4)

To a pre-dried flask flushed with argon, **Compound 3** (1 eq) was dissolved in a mixture of dichloromethane and ethanol and stirred. The mixture was sparged with argon and 5 % Pd/C catalyst was added. The argon atmosphere was evacuated under vacuum and replaced by hydrogen gas. The quantities of the reagents used in each reaction are listed in **Table 4**. The reaction was allowed to proceed for 4 h at room temperature, with the extent of the reaction monitored by TLC using an appropriate solvent system (RF values quoted in the product data). The hydrogen gas, after the reaction was completed, was evacuated under vacuum and the flask was purged using argon. The mixture was filtered through Celite, and the collected solvent was evaporated under vacuum to leave a solid which was carried forwards without any further purification.

Table 4. Quantities of reagents used in the syntheses of the 4-(2-alkoxy-4-methoxybenzoyloxy)benzoic acids

m	(**3**)	5 % Palladium on Carbon	Dichloromethane	Ethanol
2	4.20 g, 0.0103 mol	0.767 g, 7.21×10^{-3} mol	70 mL	70 mL
3	2.60 g, 6.18×10^{-3} mol	0.132 g, 1.24×10^{-3} mol	50 mL	50 mL
4	4.20 g, 9.67×10^{-3} mol	0.205 g, 1.93×10^{-3} mol	80 mL	80 mL
5	2.80 g, 6.24×10^{-3} mol	0.133 g, 1.25×10^{-3} mol	50 mL	50 mL
6	1.40 g, 3.03×10^{-3} mol	0.265 g, 2.41×10^{-3} mol	50 mL	50 mL

7	2.70 g, 5.67×10^{-3} mol	0.422 g, 3.97×10^{-3} mol	70 mL	70 mL
8	3.80 g, 7.75×10^{-3} mol	0.578 g, 5.43×10^{-3} mol	70 mL	70 mL
9	3.70 g, 7.33×10^{-3} mol	0.546 g, 5.13×10^{-3} mol	70 mL	70 mL

4.1 4-(2-Ethoxy-4-methoxybenzoyloxy)benzoic acid

White solid. Yield: 1.37 g, 42.1 %. RF: 0.095 (40 % ethyl acetate:60 % 40:60 petroleum ether). M.P = 194 °C
ν_{max}/cm^{-1}: 2995, 2836, 1705, 1683, 1604, 1573, 1505, 1429, 1388, 1315, 1301, 1268, 1249, 1213, 1176, 1139, 1110, 1074, 1044, 1033, 926, 883, 843, 822, 781, 764, 752, 738, 687, 654, 586, 551, 505, 459, 410
δ_H/ppm (400 MHz, DMSO-d$_6$): 13.01 (1 H, s, OH), 8.02 (2 H, d, J 8.6 Hz, Ar-H), 7.93 (1 H, d, J 8.7 Hz, Ar-H), 7.33 (2 H, d, J 8.6 Hz, Ar-H), 6.69 (1 H, d, J 2.2 Hz, Ar-H), 6.66 (1 H, dd, J 8.7 Hz, 2.2 Hz, Ar-H), 4.14 (2 H, q, J 7.0 Hz, O-$\underline{CH_2}$-CH_3), 3.86 (3 H, s, O-$\underline{CH_3}$), 1.34 (3 H, t, J 7.0 Hz, O-CH_2-$\underline{CH_3}$)
δ_C/ppm (100 MHz, DMSO-d$_6$): 167.16, 165.23, 163.19, 161.43, 154.78, 134.32, 131.31, 128.60, 122.65, 110.70, 106.24, 100.30, 64.73, 56.16, 14.92

4.2 4-(4-Methoxy-2-propoxybenzoyloxy)benzoic acid

White solid. Yield: 1.51 g, 74.0 %. RF: 0.088 (40 % ethyl acetate:60 % 40:60 petroleum ether). M.P = 169 °C
ν_{max}/cm^{-1}: 2973, 2840, 1745, 1681, 1603, 1568, 1503, 1428, 1387, 1318, 1295, 1243, 1197, 1164, 1139, 1046, 1006, 984, 879, 829, 760, 690, 656, 632, 619, 597, 550, 541, 512, 461, 406
δ_H/ppm (400 MHz, DMSO-d$_6$): 13.00 (1 H, br, OH), 8.02 (2 H, d, J 8.8 Hz, Ar-H), 7.93 (1 H, d, J 8.7 Hz, Ar-H), 7.32 (2 H, d, J 8.8 Hz, Ar-H), 6.68 (1 H, d, J 2.3 Hz, Ar-H), 6.66 (1 H, dd, J 8.7 Hz, 2.3 Hz, Ar-H), 4.04 (2 H, t, J 6.2 Hz, O-$\underline{CH_2}$-CH_2-), 3.86 (3 H, s, O-$\underline{CH_3}$), 1.74 (2 H, tq, J 6.2 Hz, 7.4 Hz, O-CH_2-$\underline{CH_2}$-CH_3), 0.98 (3 H, t, J 7.4 Hz, O-CH_2-CH_2-$\underline{CH_3}$)
δ_C/ppm (100 MHz, DMSO-d$_6$): 167.16, 165.29, 163.33, 161.60, 154.81, 134.40, 131.33, 128.61, 122.63, 110.55, 106.25, 100.05, 70.30, 56.16, 22.46, 10.94

4.3 4-(2-Butoxy-4-methoxybenzoyloxy)benzoic acid

Off-white solid. Yield: 2.66 g, 79.9 %. RF: 0.093 (40 % ethyl acetate:60 % 40:60 petroleum ether). M.P = 166 °C
ν_{max}/cm^{-1}: 2954, 2860, 1709, 1681, 1599, 1571, 1505, 1424, 1383, 1314, 1295, 1267, 1246, 1199, 1173, 1155, 1136, 1072, 1035, 1024, 1012, 982, 937, 876, 850, 823, 769, 759, 729, 690, 653, 588, 573, 549, 526, 507, 462, 423
δ_H/ppm (400 MHz, DMSO-d$_6$): 13.02 (1 H, br, OH), 8.02 (2 H, d, J 8.7 Hz, Ar-H), 7.93 (1 H, d, J 8.7 Hz, Ar-H), 7.32 (2 H, d, J 8.7 Hz, Ar-H), 6.70 (1 H, d, 2.3 Hz, Ar-H), 6.66 (1 H, dd, J 8.7 Hz, J 2.3 Hz, Ar-H), 4.08 (2 H, t, J 6.2 Hz, O-$\underline{CH_2}$-CH_2-), 3.86 (3 H, s, O-$\underline{CH_3}$), 1.70 (2 H, tt, J 6.2 Hz, 6.7 Hz, O-CH_2-$\underline{CH_2}$-CH_2-), 1.46 (2 H, tq, J 6.7 Hz, 7.4 Hz, O-CH_2-CH_2-$\underline{CH_2}$-CH_3), 0.89 (3 H, t, J 7.4 Hz, O-CH_2-CH_2-CH_2-$\underline{CH_3}$)
δ_C/ppm (100 MHz, DMSO-d$_6$): 167.15, 165.29, 163.29, 161.61, 154.81, 134.38, 131.32, 128.59, 122.62, 110.55, 106.23, 100.06, 68.48, 56.16, 31.09, 19.12, 14.07

4.4 4-(4-Methoxy-2-pentoxybenzoyloxy)benzoic acid

White solid. Yield: 1.80 g, 80.4 %. RF: 0.093 (40 % ethyl acetate:60 % 40:60 petroleum ether). T_{CrI} 127 °C T_{NI} (67 °C)

ν_{max}/cm^{-1}: 2956, 2855, 1749, 1682, 1604, 1571, 1505, 1429, 1293, 1241, 1202, 1173, 1155, 1048, 1008, 988, 938, 882, 827, 762, 691, 657, 602, 543, 463
δ_H/ppm (400 MHz, DMSO-d_6): 13.03 (1 H, br, OH), 8.02 (2 H, d, J 8.5 Hz, Ar-H), 7.92 (1 H, d, J 8.7 Hz, Ar-H), 7.32 (2 H, d, J 8.5 Hz, Ar-H), 6.69 (1 H, d, J 2.3 Hz, Ar-H), 6.65 (1 H, dd, J 8.7 Hz, J 2.3 Hz, Ar-H), 4.07 (2 H, t, J 6.2 Hz, O-$\underline{CH_2}$-CH_2-), 3.86 (3 H, s, O-$\underline{CH_3}$), 1.72 (2 H, tt, J 6.2 Hz, 6.9 Hz, O-CH_2-$\underline{CH_2}$-CH_2-), 1.41 (2 H, tt, J 6.9 Hz, 7.1 Hz, O-CH_2-CH_2-$\underline{CH_2}$-CH_2-), 1.29 (2 H, sept, J 7.1 Hz, O-CH_2-CH_2-CH_2-$\underline{CH_2}$-CH_3), 0.88 (3 H, t, J 7.1 Hz, O-CH_2-CH_2-CH_2-CH_2-$\underline{CH_3}$)
δ_C/ppm (100 MHz, DMSO-d_6): 167.17, 165.28, 163.42, 161.56, 154.80, 134.39, 131.30, 128.63, 122.59, 110.60, 106.24, 100.05, 68.79, 56.15, 28.73, 28.11, 22.25, 14.32

4.5 4-(2-Hexyloxy-4-methoxybenzoyloxy)benzoic acid

Off-white solid. Yield: 1.05 g, 92.7 %. RF: 0.081 (40 % ethyl acetate:60 % 40:60 petroleum ether). M.P = 146 °C
ν_{max}/cm^{-1}: 2921, 2856, 1746, 1682, 1604, 1571, 1504, 1424, 1386, 1318, 1295, 1268, 1245, 1200, 1164, 1128, 1045, 1010, 996, 935, 882, 813, 781, 765, 691, 658, 632, 618, 602, 551, 507, 462, 407
δ_H/ppm (400 MHz, DMSO-d_6): 13.03 (1 H, br, OH), 8.01 (2 H, d, J 8.7 Hz, Ar-H), 7.91 (1 H, d, J 8.7 Hz, Ar-H), 7.32 (2 H, d, J 8.7 Hz, Ar-H), 6.69 (1 H, d, J 2.3 Hz, Ar-H), 6.66 (1 H, dd, J 8.7 Hz, 2.3 Hz, Ar-H), 4.07 (2 H, t, J 6.2 Hz, O-$\underline{CH_2}$-CH_2-), 3.86 (3 H, s, O-$\underline{CH_3}$), 1.71 (2 H, tt, J 6.2 Hz, 6.8 Hz, O-CH_2-$\underline{CH_2}$-CH_2-), 1.43 (2 H, m, O-CH_2-CH_2-$\underline{CH_2}$-CH_2-), 1.24 (4 H, m, O-CH_2-CH_2-CH_2-$\underline{CH_2}$-$\underline{CH_2}$-CH_3), 0.79 (3 H, t, J 7.0 Hz, O-CH_2-CH_2-CH_2-CH_2-CH_2-$\underline{CH_3}$)
δ_C/ppm (100 MHz, DMSO-d_6): 167.16, 165.28, 163.47, 161.55, 154.83, 134.39, 131.31, 128.55, 122.60, 110.61, 106.26, 100.06, 68.82, 56.17, 31.37, 29.02, 25.60, 22.50, 14.28

4.6 4-(2-Heptyloxy-4-methoxybenzoyloxy)benzoic acid

White solid. Yield: 1.85 g, 84.4 %. RF: 0.382 (100 % ethyl acetate). M.P = 141 °C
ν_{max}/cm^{-1}: 2925, 2856, 1749, 1682, 1605, 1571, 1504, 1462, 1430, 1383, 1292, 1264, 1242, 1203, 1163, 1148, 1127, 1046, 1004, 963, 885, 830, 779, 762, 730, 962, 657, 620, 603, 544, 504, 412
δ_H/ppm (400 MHz, DMSO-d_6): 13.01 (1 H, br, OH), 8.01 (2 H, d, J 8.6 Hz, Ar-H), 7.91 (1 H, d, J 8.7 Hz, Ar-H), 7.31 (2 H, d, J 8.6 Hz, Ar-H), 6.69 (1 H, d, J 2.3 Hz, Ar-H), 6.65 (1 H, dd, J 8.7 Hz, 2.3 Hz, Ar-H), 4.07 (2 H, t, J 6.1 Hz, O-$\underline{CH_2}$-CH_2-), 3.86 (3 H, s, O-$\underline{CH_3}$), 1.71 (2 H, tt, J 6.1 Hz, 6.8 Hz, O-CH_2-$\underline{CH_2}$-CH_2-), 1.41 (2 H, m, O-CH_2-CH_2-$\underline{CH_2}$-CH_2-), 1.20 (6 H, m, O-CH_2-CH_2-CH_2-$\underline{CH_2}$-$\underline{CH_2}$-$\underline{CH_2}$-CH_3), 0.79 (3 H, t, J 6.9 Hz, O-CH_2-CH_2-CH_2-CH_2-CH_2-CH_2-$\underline{CH_3}$)
δ_C/ppm (100 MHz, DMSO-d_6): 167.15, 165.28, 163.53, 161.53, 154.82, 134.38, 131.29, 128.59, 122.56, 110.63, 106.26, 100.06, 68.81, 56.16, 31.68, 29.08, 28.85, 25.91, 22.44, 14.36

4.7 4-(4-Methoxy-2-octyloxybenzoyloxy)benzoic acid

White solid. Yield: 2.53 g, 81.5 %. RF: 0.382 (100 % ethyl acetate). M.P = 139 °C
ν_{max}/cm^{-1}: 2926, 2851, 1750, 1683, 1605, 1573, 1505, 1425, 1388, 1293, 1262, 1242, 1205, 1165, 1149, 1128, 1050, 1012, 970, 884, 833, 763, 693, 658, 603, 554, 503, 469, 431, 421, 404
δ_H/ppm (400 MHz, DMSO-d_6): 13.01 (1 H, br, OH), 8.01 (2 H, d, J 8.7 Hz, Ar-H), 7.91 (1 H, d, J 8.7 Hz, Ar-H), 7.31 (2 H, d, J 8.7 Hz, Ar-H), 6.68 (1 H, d, J 2.3 Hz, Ar-H), 6.65 (1 H, dd, J 8.7 Hz, 2.3 Hz, Ar-H), 4.07 (2 H, t, J 6.1 Hz, O-$\underline{CH_2}$-CH_2-), 3.86 (3 H, s, O-$\underline{CH_3}$), 1.70 (2 H, tt, J 6.1 Hz, 6.7 Hz, O-CH_2-$\underline{CH_2}$-CH_2-), 1.42 (2 H, tt, J 6.7 Hz, 7.0 Hz, O-CH_2-CH_2-$\underline{CH_2}$-CH_2-), 1.18 (8 H, m, O-CH_2-CH_2-CH_2-$\underline{CH_2}$-$\underline{CH_2}$-$\underline{CH_2}$-$\underline{CH_2}$-CH_3), 0.79 (3 H, t, J 7.0 Hz, O-CH_2-CH_2-CH_2-CH_2-CH_2-CH_2-CH_2-$\underline{CH_3}$)

δ_C/ppm (100 MHz, DMSO-d$_6$): 167.13, 165.27, 163.55, 161.52, 154.82, 134.38, 131.29, 128.57, 122.55, 110.64, 106.25, 100.05, 68.81, 56.15, 31.63, 29.16, 29.12, 29.08, 25.98, 22.52, 14.35

4.8 4-(4-Methoxy-2-nonyloxybenzoyloxy)benzoic acid

White solid. Yield: 2.67 g, 87.9 %. RF: 0.411 (100 % ethyl acetate). M.P = 137 °C

v_{max}/cm^{-1}: 2950, 2922, 2848, 1748, 1682, 1605, 1572, 1505, 1462, 1427, 1388, 131, 1293, 1261, 1243, 1202, 1174, 1163, 1147, 1128, 1048, 1020, 1007, 976, 942, 887, 832, 814, 779, 762, 726, 692, 657, 619, 602, 552, 554, 505, 419

δ_H/ppm (400 MHz, DMSO-d$_6$): 13.00 (1 H, br, OH), 8.01 (2 H, d, J 8.7 Hz, Ar-H), 7.90 (1 H, d, J 8.7 Hz, Ar-H), 7.31 (2 H, d, J 8.7 Hz, Ar-H), 6.68 (1 H, d, J 2.3 Hz, Ar-H), 6.65 (1 H, dd, J 8.7 Hz, 2.3 Hz, Ar-H), 4.07 (2 H, t, J 6.1 Hz, O-$\underline{CH_2}$-CH_2-), 3.86 (3 H, s, O-$\underline{CH_3}$), 1.70 (2 H, tt, J 6.1 Hz, 6.8 Hz, O-CH_2-$\underline{CH_2}$-CH_2-), 1.42 (2 H, m, O-CH_2-CH_2-$\underline{CH_2}$-CH_2-), 1.21 (10 H, m, O-CH_2-CH_2-CH_2-$\underline{CH_2}$-$\underline{CH_2}$-$\underline{CH_2}$-$\underline{CH_2}$-$\underline{CH_2}$-CH_3), 0.81 (3 H, t, J 7.0 Hz, O-CH_2-CH_2-CH_2-CH_2-CH_2-CH_2-CH_2-CH_2-$\underline{CH_3}$)

δ_C/ppm (100 MHz, DMSO-d$_6$): 167.15, 165.27, 163.57, 161.51, 154.81, 134.39, 131.28, 128.62, 122.54, 110.64, 106.26, 100.05, 68.81, 56.16, 31.73, 29.41, 29.19, 29.08, 29.04, 25.97, 22.51, 14.38

4-[(4-Nitrophenoxy)carbonyl]phenyl 2-alkoxy-4-methoxybenzoates (5-m)

To a pre-dried flask flushed with argon and kept in an ice bath in order to maintain the temperature at 0°C, **Compound 4** (1 eq), 4-nitrophenol (1.3 eq, 0.91 eq for *m* = 4, 6) and 4-dimethylaminopyridine (0.15 eq, 0.1 eq for *m* = 4, 6) were added. The solids were solubilised with dichloromethane (30 mL) and stirred for 10 min before *N*-ethyl-*N′*-(3-dimethylaminopropyl)carbodiimide hydrochloride (1.5 eq) (*N,N′*-dicyclohexylcarbodiimide (1.2 eq) for *m* = 4, 6) was added to the flask. The quantities of the reagents used in each reaction are listed in **Table 5**. The temperature of the reaction mixture was increased to room temperature and the reaction was allowed to proceed overnight. For *m* = 4, 6, the white precipitate which formed was removed by vacuum filtration and the filtrate collected. The solvent was removed under vacuum and the crude product was purified using a silica gel column with an appropriate solvent system (RF values quoted in product data). The eluent fractions of interest were evaporated under vacuum to leave a white solid which was recrystallised from hot ethanol (50 mL).

Table 5. Quantities of reagents used in the syntheses of the 4-[(4-nitrophenoxy)carbonyl]phenyl 2-alkoxy-4-methoxybenzoates

m	(**4**)	4-Nitrophenol	4-Dimethylaminopyridine	*N*-Ethyl-*N′*-(3-dimethylaminopropyl)carbodiimide Hydrochloride/*N,N′*-Dicyclohexylcarbodiimide
2	0.300 g, 9.48×10^{-4} mol	0.171 g, 1.23×10^{-3} mol	0.0173 g, 1.42×10^{-4} mol	0.272 g, 1.42×10^{-3} mol
3	0.300 g, 9.08×10^{-4}	0.164 g, 1.18×10^{-3}	0.0166 g, 1.36×10^{-4} mol	0.261 g, 1.36×10^{-3} mol

	mol	mol		
4	0.300 g, $8.71{\times}10^{-4}$ mol	0.110 g, $7.92{\times}10^{-4}$ mol	0.0106 g, $8.71{\times}10^{-5}$ mol	0.196 g, $9.50{\times}10^{-4}$ mol
5	0.300 g, $8.37{\times}10^{-4}$ mol	0.152 g, $1.09{\times}10^{-3}$ mol	0.0154 g, $1.26{\times}10^{-4}$ mol	0.242 g, $1.26{\times}10^{-3}$ mol
6	0.300 g, $8.06{\times}10^{-4}$ mol	0.101 g, $7.33{\times}10^{-4}$ mol	$8.96{\times}10^{-3}$ g, $7.33{\times}10^{-5}$ mol	0.181 g, $8.89{\times}10^{-4}$ mol
7	0.300 g, $7.76{\times}10^{-4}$ mol	0.141 g, $1.01{\times}10^{-3}$ mol	0.0142 g, $1.16{\times}10^{-4}$ mol	0.222 g, $1.16{\times}10^{-3}$ mol
8	0.300 g, $7.49{\times}10^{-4}$ mol	0.135 g, $9.74{\times}10^{-4}$ mol	0.0137 g, $1.12{\times}10^{-4}$ mol	0.215 g, $1.12{\times}10^{-3}$ mol
9	0.300 g, $7.24{\times}10^{-4}$ mol	0.131 g, $9.41{\times}10^{-3}$ mol	0.0133 g, $1.09{\times}10^{-4}$ mol	0.209 g, $1.09{\times}10^{-3}$ mol

5.1 4-[(4-Nitrophenoxy)carbonyl]phenyl 2-ethoxy-4-methoxybenzoate (5-2)

Yield: 0.090 g, 21.7 %. RF: 0.606 (10 % ethyl acetate:90 % dichloromethane).

T_{CrI} 159 °C T_{N_FN} (106 °C) T_{NI} (131 °C)

ν_{max}/cm^{-1}: 3083, 2989, 1752, 1734, 1610, 1567, 1517, 1493, 1433, 1414, 1389, 1352, 1308, 1265, 1247, 1199, 1161, 1157, 1143, 1110, 1062, 1036, 994, 955, 884, 863, 845, 821, 801, 757, 745, 683, 671, 635, 618, 595, 544, 511, 500, 482, 412

δ_H/ppm (400 MHz, DMSO-d_6): 8.37 (2 H, d, J 9.0 Hz, Ar-H), 8.24 (2 H, d, J 8.6 Hz, Ar-H), 7.97 (1 H, d, J 8.6 Hz, Ar-H), 7.65 (2 H, d, J 9.0 Hz, Ar-H), 7.49 (2 H, d, J 8.6 Hz, Ar-H), 6.71 (1 H, d, J 2.2 Hz, Ar-H), 6.68 (1 H, dd, J 8.6 Hz, 2.2 Hz, Ar-H), 4.16 (2 H, q, J 7.0 Hz, O-$\underline{CH_2}$-CH_3), 3.87 (3 H, s, O-$\underline{CH_3}$), 1.36 (3 H, t, J 7.0 Hz, O-CH_2-$\underline{CH_3}$)

δ_C/ppm (100 MHz, DMSO-d_6): 165.36, 163.77, 163.01, 161.54, 155.97, 155.96, 145.67, 134.44, 132.22, 126.03, 125.79, 123.86, 123.19, 110.49, 106.31, 100.31, 64.76, 56.19, 14.93

MS = $[M+H]^+$: Calculated for $C_{23}H_{20}NO_8$: 438.1189. Found: 438.1195. Difference: 1.4 ppm

5.2 4-[(4-Nitrophenoxy)carbonyl]phenyl 4-methoxy-2-propoxybenzoate (5-3)

Yield: 0.074 g, 18.1 %. RF: 0.242 (100 % dichloromethane).

T_{CrI} 147 °C T_{N_FN} (85 °C) T_{NI} (97 °C)

ν_{max}/cm^{-1}: 3058, 2948, 1738, 1717, 1610, 1600, 1584, 1528, 1508, 1487, 1442, 1428, 1399, 1349, 1331, 1294, 1270, 1217, 1207, 1181, 1160, 1150, 1119, 1094, 1069, 1049, 1021, 1014, 967, 958, 887, 854, 841, 816, 767, 751, 687, 667, 630, 588, 540, 529, 507, 472, 458, 409

δ_H/ppm (400 MHz, DMSO-d_6): 8.36 (2 H, d, J 9.1 Hz, Ar-H), 8.24 (2 H, d, J 8.8 Hz, Ar-H), 7.96 (1 H, d, J 8.7 Hz, Ar-H), 7.65 (2 H, d, J 9.1 Hz, Ar-H), 7.47 (2 H, d, J 8.8 Hz, Ar-H), 6.70 (1 H, d, J 2.3 Hz, Ar-H), 6.67 (1 H, dd, J 8.7 Hz, 2.3 Hz, Ar-H), 4.06 (2 H, t, J 6.3 Hz, O-$\underline{CH_2}$-CH_2-), 3.87 (3 H, s, O-$\underline{CH_3}$), 1.76 (2 H, tq, J 6.3 Hz, 7.2 Hz, O-CH_2-$\underline{CH_2}$-CH_3), 1.00 (3 H, t, J 7.2 Hz, O-CH_2-CH_2-$\underline{CH_3}$)

δ_C/ppm (100 MHz, DMSO-d_6): 165.41, 163.76, 163.18, 161.70, 155.99, 155.97, 145.66, 134.51, 132.23, 126.03, 125.79, 123.85, 123.17, 110.34, 106.32, 100.07, 70.33, 56.19, 22.48, 10.95

MS = $[M+Na]^+$: Calculated for $C_{24}H_{21}NO_8Na$: 474.1165. Found: 474.1150. Difference: 3.2 ppm

5.3 4-[(4-Nitrophenoxy)carbonyl]phenyl 2-butoxy-4-methoxybenzoate (5-4)

Yield: 0.050 g, 12.3 %. RF: 0.306 (20 % ethyl acetate:80 % 40:60 petroleum ether).

T_{CrI} 141 °C T_{N_FN} (65 °C) T_{NI} (75 °C)

ν_{max}/cm^{-1}: 2966, 1741, 1691, 1592, 1574, 1510, 1486, 1466, 1448, 1425, 1413, 1393, 1336, 1295, 1258, 1241, 1207, 1195, 1160, 1111, 1078, 1054, 1028, 1014, 1009, 887, 862, 842, 827, 820, 767, 756, 743, 688, 677, 655, 627, 595, 529, 509, 472, 408

δ_H/ppm (400 MHz, DMSO-d_6): 8.36 (2 H, d, J 8.9 Hz, Ar-H), 8.25 (2 H, d, J 8.6 Hz, Ar-H), 7.97 (1 H, d, J 8.7 Hz, Ar-H), 7.66 (2 H, d, J 8.9 Hz, Ar-H), 7.47 (2 H, d, J 8.6 Hz, Ar-H), 6.71 (1 H, d, J 2.2 Hz, Ar-H), 6.67 (1 H, dd, J 8.7 Hz, 2.2 Hz, Ar-H), 4.11 (2 H, t, J 6.2 Hz, O-$\underline{CH_2}$-CH_2-), 3.88 (3 H, s, O-$\underline{CH_3}$), 1.70 (2 H, tt, J 6.2 Hz, 6.9 Hz, O-CH_2-$\underline{CH_2}$-CH_2-), 1.48 (2 H, tq, J 6.9 Hz, 7.4 Hz, O-CH_2-CH_2-$\underline{CH_2}$-CH_3), 0.91 (3 H, t, J 7.4 Hz, O-CH_2-CH_2-CH_2-$\underline{CH_3}$)

δ_C/ppm (100 MHz, DMSO-d_6): 165.43, 163.79, 163.18, 161.73, 156.01, 155.99, 145.69, 134.52, 132.24, 126.05, 125.81, 123.88, 123.18, 110.35, 106.34, 100.09, 68.52, 56.21, 31.09, 19.12, 14.08

MS = $[M+Na]^+$: Calculated for $C_{25}H_{23}NO_8Na$: 488.1321. Found: 488.1344. Difference: 4.7 ppm

5.4 4-[(4-Nitrophenoxy)carbonyl]phenyl 4-methoxy-2-pentoxybenzoate (5-5)

Yield: 0.147 g, 36.6 %. RF: 0.667 (4 % ethyl acetate:96 % dichloromethane).

T_{CrI} 132 °C T_{N_FN} (54 °C) T_{NI} (61 °C)

ν_{max}/cm^{-1}: 2934, 1741, 1689, 1601, 1592, 1575, 1523, 1510, 1489, 1471, 1447, 1425, 1414, 1395, 1341, 1296, 1259, 1239, 1216, 1194, 1160, 1114, 1077, 1052, 1031, 1012, 991, 887, 862, 829, 768, 756, 744, 688, 667, 656, 628, 595, 527, 511, 500, 472, 409

δ_H/ppm (400 MHz, DMSO-d_6): 8.36 (2 H, d, J 9.1 Hz, Ar-H), 8.24 (2 H, d, J 8.6 Hz, Ar-H), 7.95 (1 H, d, J 8.7 Hz, Ar-H), 7.65 (2 H, d, J 9.1 Hz, Ar-H), 7.46 (2 H, d, J 8.6 Hz, Ar-H), 6.70 (1 H, d, J 2.3 Hz, Ar-H), 6.67 (1 H, dd, J 8.7 Hz, 2.3 Hz, Ar-H), 4.09 (2 H, t, J 6.2 Hz, O-$\underline{CH_2}$-CH_2-), 3.87 (3 H, s, O-$\underline{CH_3}$), 1.73 (2 H, tt, J 6.2 Hz, 6.7 Hz, O-CH_2-$\underline{CH_2}$-CH_2-), 1.43 (2 H, tt, J 6.7 Hz, 7.2 Hz, O-CH_2-CH_2-$\underline{CH_2}$-CH_2-), 1.30 (2 H, sept, J 7.2 Hz, O-CH_2-CH_2-CH_2-$\underline{CH_2}$-CH_3), 0.83 (3 H, t, J 7.2 Hz, O-CH_2-CH_2-CH_2-CH_2-$\underline{CH_3}$)

δ_C/ppm (100 MHz, DMSO-d_6): 165.41, 163.76, 163.25, 161.67, 156.00, 155.96, 145.66, 134.51, 132.21, 126.02, 125.78, 123.85, 123.16, 110.36, 106.31, 100.06, 68.82, 56.19, 28.71, 28.12, 22.25, 14.36

MS = $[M+H]^+$: Calculated for $C_{26}H_{26}NO_8$: 480.1658. Found: 480.1669. Difference: 2.3 ppm

5.5 4-[(4-Nitrophenoxy)carbonyl]phenyl 2-hexyloxy-4-methoxybenzoate (5-6)

Yield: 0.052 g, 14.4 %. RF: 0.333 (20 % ethyl acetate: 80 % 40:60 petroleum ether).

T_{CrI} 101 °C T_{N_FN} (44 °C) T_{NI} (50 °C)

ν_{max}/cm^{-1}: 2933, 1742, 1732, 1605, 1594, 1570, 1528, 1504, 1489, 1474, 1441, 1429, 1390, 1345, 1298, 1268, 1246, 1197, 1167, 1141, 1065, 1050, 1038, 1009, 993, 891, 863, 844, 803, 756, 742, 732, 693, 668, 630, 615 ,600, 498, 482, 471, 407

δ_H/ppm (400 MHz, DMSO-d_6): 8.36 (2 H, d, J 9.1 Hz, Ar-H), 8.24 (2 H, d, J 8.6 Hz, Ar-H), 7.95 (1 H, d, J 8.7 Hz, Ar-H), 7.65 (2 H, d, J 9.1 Hz, Ar-H), 7.46 (2 H, d, J 8.6 Hz, Ar-H), 6.70 (1 H, d, J 2.3 Hz, Ar-H), 6.67 (1 H, dd, J 8.7 Hz, 2.3 Hz, Ar-H), 4.10 (2 H, t, J 6.2 Hz, O-$\underline{CH_2}$-CH_2-), 3.88 (3 H, s, O-$\underline{CH_3}$), 1.73 (2 H, tt, J 6.2 Hz, 6.8 Hz, O-CH_2-$\underline{CH_2}$-CH_2-), 1.45 (2 H, quin, J 6.8 Hz, O-CH_2-CH_2-$\underline{CH_2}$-CH_2-), 1.25 (4 H, m, O-CH_2-CH_2-CH_2-$\underline{CH_2\text{-}CH_2}$-$CH_3$), 0.82 (3 H, t, J 6.8 Hz, O-CH_2-CH_2-CH_2-CH_2-CH_2-$\underline{CH_3}$)

δ_C/ppm (100 MHz, DMSO-d_6): 165.42, 163.79, 163.29, 161.68, 156.02, 155.97, 145.69, 134.52, 132.22, 126.03, 125.81, 123.86, 123.16, 110.40, 106.34, 100.08, 68.85, 56.20, 31.35, 29.00, 25.59, 22.51, 14.31
MS = $[M+Na]^+$: Calculated for $C_{27}H_{27}NO_8Na$: 516.1634. Found: 516.1635. Difference: 0.2 ppm

5.6 4-[(4-Nitrophenoxy)carbonyl]phenyl 2-heptyloxy-4-methoxybenzoate (5-7)

Yield: 0.026 g, 6.60 %. RF: 0.108 (10 % ethyl acetate: 90 % 40:60 petroleum ether).
T_{CrI} 110 °C T_{N_FN} (44 °C) T_{NI} (50 °C)
ν_{max}/cm^{-1}: 2932, 1732, 1605, 1571, 1530, 1503, 1490, 1472, 1453, 1441, 1430, 1390, 1346, 1325, 1297, 1268, 1246, 1195, 116, 1141, 1065, 1050, 1003, 892, 868, 841, 822, 804, 757, 742, 726, 692, 669, 629, 615, 601, 548, 529, 508, 482, 418
δ_H/ppm (400 MHz, DMSO-d_6): 8.37 (2 H, d, J 9.1 Hz, Ar-H), 8.25 (2 H, d, J 8.7 Hz, Ar-H), 7.95 (1 H, d, J 8.7 Hz, Ar-H), 7.64 (2 H, d, J 9.1 Hz, Ar-H), 7.47 (2 H, d, J 8.7 Hz, Ar-H), 6.71 (1 H, d, J 2.3 Hz, Ar-H), 6.67 (1 H, dd, J 8.7 Hz, 2.3 Hz, Ar-H), 4.09 (2 H, t, J 6.2 Hz, O-$\underline{CH_2}$-CH_2-), 3.87 (3 H, s, O-$\underline{CH_3}$), 1.73 (2 H, tt, J 6.2 Hz, 6.8 Hz, O-CH_2-$\underline{CH_2}$-CH_2-), 1.41 (2 H, m, O-CH_2-CH_2-$\underline{CH_2}$-CH_2-), 1.22 (6 H, m, O-CH_2-CH_2-CH_2-$\underline{CH_2}$-$\underline{CH_2}$-$\underline{CH_2}$-CH_3), 0.82 (3 H, t, J 7.1 Hz, O-CH_2-CH_2-CH_2-CH_2-CH_2-CH_2-$\underline{CH_3}$)
δ_C/ppm (100 MHz, DMSO-d_6): 165.40, 163.77, 163.33, 161.65, 156.01, 155.95, 145.67, 134.50, 132.21, 126.00, 125.81, 123.84, 123.14, 110.40, 106.34, 100.08, 68.83, 56.20, 31.69, 29.07, 28.84, 25.90, 22.47, 14.39
MS = $[M+Na]^+$: Calculated for $C_{28}H_{29}NO_8Na$: 530.1791. Found: 530.1791. Difference: 0 ppm

5.7 4-[(4-Nitrophenoxy)carbonyl]phenyl 4-methoxy-2-octyloxybenzoate (5-8)

Yield: 0.056 g, 14.3 %. RF: 0.342 (100 % dichloromethane).
T_{CrI} 123 °C T_{N_FN} (44 °C) T_{NI} (51 °C)
ν_{max}/cm^{-1}: 2929, 2856, 1732, 1605, 1571, 1527, 1504, 1490, 1469, 1441, 1434, 1429, 1390, 1346, 1298, 1267, 1245, 1196, 1167, 1141, 1066, 1049, 1008, 969, 892, 868, 841, 803, 756, 743, 723, 692, 668, 629, 615, 601, 547, 507, 482, 412
δ_H/ppm (400 MHz, DMSO-d_6): 8.36 (2 H, d, J 9.2 Hz, Ar-H), 8.24 (2 H, d, J 8.8 Hz, Ar-H), 7.94 (1 H, d, J 8.7 Hz, Ar-H), 7.64 (2 H, d, J 9.2 Hz, Ar-H), 7.46 (2 H, d, J 8.8 Hz, Ar-H), 6.70 (1 H, d, J 2.3 Hz, Ar-H), 6.67 (1 H, dd, J 8.7 Hz, 2.3 Hz, Ar-H), 4.08 (2 H, t, J 6.1 Hz, O-$\underline{CH_2}$-CH_2-), 3.87 (3 H, s, O-$\underline{CH_3}$), 1.72 (2 H, tt, J 6.1 Hz, 6.9 Hz, O-CH_2-$\underline{CH_2}$-CH_2-), 1.42 (2 H, tt, J 6.9 Hz, 7.1 Hz, O-CH_2-CH_2-$\underline{CH_2}$-CH_2-), 1.20 (8 H, m, O-CH_2-CH_2-CH_2-$\underline{CH_2}$-$\underline{CH_2}$-$\underline{CH_2}$-$\underline{CH_2}$-CH_3), 0.80 (3 H, t, J 7.1 Hz, O-CH_2-CH_2-CH_2-CH_2-CH_2-CH_2-CH_2-$\underline{CH_3}$)
δ_C/ppm (100 MHz, DMSO-d_6): 165.40, 163.75, 163.36, 161.64, 156.02, 155.94, 145.66, 134.50, 132.20, 125.99, 125.80, 123.81, 123.12, 110.41, 106.32, 100.07, 68.84, 56.19, 31.65, 29.17, 29.14, 29.08, 25.97, 22.54, 14.38
MS = $[M+H]^+$: Calculated for $C_{29}H_{32}NO_8$: 522.2128. Found: 522.2125. Difference: 0.6 ppm

5.8 4-[(4-Nitrophenoxy)carbonyl]phenyl 4-methoxy-2-nonyloxybenzoate (5-9)

Yield: 0.075 g, 19.3 %. RF: 0.371 (100 % dichloromethane).
T_{CrI} 116 °C T_{N_FN} (42 °C) T_{NI} (49 °C)
ν_{max}/cm^{-1}: 2919, 2854, 1732, 1605, 1572, 1526, 1504, 1469, 1441, 1429, 1391, 1346, 1297, 1269, 1245, 1196, 1166, 1141, 1065, 1049, 1020, 1007, 976, 893, 868, 840, 803, 756, 743, 720, 692, 668, 629, 615, 601, 548, 506, 483, 412
δ_H/ppm (400 MHz, DMSO-d_6): 8.37 (2 H, d, J 9.1 Hz, Ar-H), 8.24 (2 H, d, J 8.7 Hz, Ar-H), 7.94 (1 H, d, J 8.7 Hz, Ar-H), 7.63 (2 H, d, J 9.1 Hz, Ar-H), 7.46 (2 H, d, J 8.7 Hz, Ar-H), 6.70 (1 H, d, J

2.3 Hz, Ar-H), 6.66 (1 H, dd, J 8.7 Hz, 2.3 Hz, Ar-H), 4.08 (2 H, t, J 6.1 Hz, O-$\underline{CH_2}$-CH_2-), 3.87 (3 H, s, O-$\underline{CH_3}$), 1.72 (2 H, tt, J 6.1 Hz, 7.0 Hz, O-CH_2-$\underline{CH_2}$-CH_2-), 1.43 (2 H, m, O-CH_2-CH_2-$\underline{CH_2}$-CH_2-), 1.19 (10 H, m, O-CH_2-CH_2-CH_2-$\underline{CH_2}$-$\underline{CH_2}$-$\underline{CH_2}$-$\underline{CH_2}$-$\underline{CH_2}$-CH_3), 0.80 (3 H, t, J 7.1 Hz, O-CH_2-CH_2-CH_2-CH_2-CH_2-CH_2-CH_2-CH_2-$\underline{CH_3}$)
δ_C/ppm (100 MHz, DMSO-d_6): 165.40, 163.74, 163.37, 161.64, 156.02, 155.94, 145.66, 134.50, 132.19, 125.99, 125.80, 123.79, 123.13, 110.41, 106.33, 100.06, 68.83, 56.49, 31.74, 29.42, 29.18, 29.06, 25.96, 22.52, 19.01, 14.38
MS = $[M+Na]^+$: Calculated for $C_{30}H_{33}NO_8Na$: 558.2104. Found: 558.2130. Difference: 4.7 ppm

4-[(3-Fluoro-4-nitrophenoxy)carbonyl]phenyl 2-alkoxy-4-methoxybenzoates (6)

To a pre-dried flask flushed with argon and kept in an ice bath in order to maintain the temperature at 0°C, **Compound 4** (1 eq), 3-fluoro-4-nitrophenol (1.3 eq, 0.91 eq for *m* = 6) and 4-dimethylaminopyridine (0.15 eq, 0.1 eq for *m* = 6) were added. The solids were solubilised with dichloromethane (30 mL) and stirred for 10 min before *N*-ethyl-*N′*-(3-dimethylaminopropyl)carbodiimide hydrochloride (1.5 eq) (*N,N′*-dicyclohexylcarbodiimide (1.2 eq) for *m* = 6) was added to the flask. The quantities of the reagents used in each reaction are listed in **Table 6**. The temperature of the reaction mixture was increased to room temperature and the reaction was allowed to proceed overnight. For *m* = 6, the off-white precipitate which formed was removed by vacuum filtration and the filtrate collected. The solvent was removed under vacuum and the crude product was purified using a silica gel column with an appropriate solvent system (RF values quoted in product data). The eluent fractions of interest were evaporated under vacuum to leave a white solid which was recrystallised from hot ethanol (50 mL).

Table 6. Quantities of reagents used in the syntheses of the 4-[(3-fluoro-4-nitrophenoxy)carbonyl]phenyl 2-alkoxy-4-methoxybenzoates

m	(**4**)	3-Fluoro-4-nitrophenol	4-Dimethylaminopyridine	*N*-Ethyl-*N′*-(3-dimethylaminopropyl)carbodiimide Hydrochloride/*N,N′*-Dicyclohexylcarbodiimide
2	0.300 g, 9.48×10^{-4} mol	0.171 g, 1.23×10^{-3} mol	0.0173 g, 1.42×10^{-4} mol	0.272 g, 1.42×10^{-3} mol
3	0.300 g, 9.08×10^{-4} mol	0.164 g, 1.18×10^{-3} mol	0.0166 g, 1.36×10^{-4} mol	0.261 g, 1.36×10^{-3} mol
4	0.300 g, 8.71×10^{-4} mol	0.110 g, 7.92×10^{-4} mol	0.0106 g, 8.71×10^{-5} mol	0.196 g, 9.50×10^{-4} mol
5	0.300 g, 8.37×10^{-4}	0.152 g, 1.09×10^{-3}	0.0154 g, 1.26×10^{-4} mol	0.242 g, 1.26×10^{-3} mol

	mol	mol		
6	0.300 g, 8.06×10^{-4} mol	0.101 g, 7.33×10^{-4} mol	8.96×10^{-3} g, 7.33×10^{-5} mol	0.181 g, 8.89×10^{-4} mol
7	0.300 g, 7.76×10^{-4} mol	0.141 g, 1.01×10^{-3} mol	0.0142 g, 1.16×10^{-4} mol	0.222 g, 1.16×10^{-3} mol
8	0.300 g, 7.49×10^{-4} mol	0.135 g, 9.74×10^{-4} mol	0.0137 g, 1.12×10^{-4} mol	0.215 g, 1.12×10^{-3} mol
9	0.300 g, 7.24×10^{-4} mol	0.131 g, 9.41×10^{-3} mol	0.0133 g, 1.09×10^{-4} mol	0.209 g, 1.09×10^{-3} mol

6.1 4-[(3-Fluoro-4-nitrophenoxy)carbonyl]phenyl 2-ethoxy-4-methoxybenzoate (6-2)

Yield: 0.123 g, 28.5 %. RF: 0.757 (100 % ethyl acetate).

T_{CrI} 160 °C T_{N_FI} (109 °C)

ν_{max}/cm^{-1}: 3057, 1761, 1733, 1608, 1581, 1531, 1509, 1488, 1443, 1415, 1350, 1331, 1298, 1255, 1234, 1207, 1181, 1160, 1130, 1094, 1054, 1037, 1005, 981, 895, 871, 841, 813, 756, 749, 729, 680, 632, 608, 576, 540, 507, 487, 462

δ_H/ppm (400 MHz, DMSO-d_6): 8.32 (1 H, t, J 8.9 Hz, Ar-H), 8.23 (2 H, d, J 8.6 Hz, Ar-H), 7.97 (1 H, d, J 8.7 Hz, Ar-H), 7.79 (1 H, dd, J 12.0 Hz, 2.3 Hz, Ar-H), 7.49 (3 H, m, Ar-H), 6.71 (1 H, d, J 2.2 Hz, Ar-H), 6.68 (1 H, dd, J 8.7 Hz, 2.2 Hz, Ar-H), 4.16 (2 H, q, J 6.9 Hz, O-$\underline{CH_2}$-CH_3), 3.87 (3 H, s, O-$\underline{CH_3}$), 1.35 (3 H, t, J 6.9 Hz, O-CH_2-$\underline{CH_3}$)

δ_F/ppm (376 MHz, DMSO-d_6): -115.38

δ_C/ppm (100 MHz, DMSO-d_6): 165.37, 163.45, 162.98, 161.55, 156.99, 156.10, 156.06, 155.99, 154.38, 135.25, 135.17, 134.45, 132.28, 128.00, 125.76, 123.23, 119.73, 119.69, 113.44, 113.20, 110.45, 106.31, 100.30, 64.76, 56.19, 14.93

MS = $[M+Na]^+$: Calculated for $C_{23}H_{18}NO_8FNa$: 478.0914. Found: 478.0935. Difference: 4.4 ppm

6.2 4-[(3-Fluoro-4-nitrophenoxy)carbonyl]phenyl 4-methoxy-2-propoxybenzoate (6-3)

Yield: 0.113 g, 26.5 %. RF: 0.286 (100 % dichloromethane).

T_{CrI} 129 °C T_{N_FI} (88 °C)

ν_{max}/cm^{-1}: 3058, 2947, 2948, 1738, 1717, 1610, 1600, 1584, 1528, 1508, 1481, 1471, 1442, 1428, 1415, 1393, 1349, 1331, 1294, 1270, 1239, 1217, 1207, 1181, 1160, 1150, 1119, 1094, 1069, 1049, 1021, 1014, 967, 958, 911, 887, 841, 816, 766, 751, 868, 667, 630, 588, 540, 529, 507, 472, 458, 432, 409

δ_H/ppm (400 MHz, DMSO-d_6): 8.32 (1 H, t, J 8.9 Hz, Ar-H), 8.23 (2 H, d, J 8.8 Hz, Ar-H), 7.96 (1 H, d, J 8.7 Hz, Ar-H), 7.78 (1 H, dd, J 12.0 Hz, 2.4 Hz, Ar-H), 7.48 (3 H, m, Ar-H), 6.70 (1 H, d, J 2.3 Hz, Ar-H), 6.67 (1 H, dd, J 8.7 Hz, 2.3 Hz, Ar-H), 4.06 (2 H, t, J 6.2 Hz, O-$\underline{CH_2}$-CH_2-), 3.87 (3 H, s, O-$\underline{CH_3}$), 1.75 (2 H, tq, J 6.2 Hz, 7.4 Hz, O-CH_2-$\underline{CH_2}$-CH_3), 1.00 (3 H, t, J 7.4 Hz, O-CH_2-CH_2-$\underline{CH_3}$)

δ_F/ppm (376 MHz, DMSO-d_6): -115.36

δ_C/ppm (100 MHz, DMSO-d_6): 165.42, 163.43, 163.15, 161.71, 156.99, 156.09, 155.99, 154.38, 135.23, 135.16, 134.52, 132.30, 128.01, 127.99, 125.76, 123.19, 119.71, 119.68, 113.42, 113.19, 110.32, 106.32, 100.06, 70.33, 56.19, 22.47, 10.94

MS = $[M+Na]^+$: Calculated for $C_{24}H_{20}NO_8FNa$: 492.1071. Found: 492.1083. Difference: 2.4 ppm

6.3 4-[(3-Fluoro-4-nitrophenoxy)carbonyl]phenyl 2-butoxy-4-methoxybenzoate (6-4)

Yield: 0.055 g, 13.1 %. RF: 0.368 (100 % dichloromethane).

T_{CrI} 119 °C T_{N_FI} (69 °C)

ν_{max}/cm^{-1}: 2964, 1746, 1693, 1600, 1575, 1529, 1508, 1484, 1448, 1426, 1414, 1394, 1346, 1335, 1295, 1234, 1215, 1197, 1155, 1139, 1113, 1093, 1072, 1048, 1029, 1014, 898, 964, 886, 842, 828, 807, 767 748, 688, 669, 654, 632, 606, 595, 584, 539, 524, 508, 459, 410

δ_H/ppm (400 MHz, DMSO-d_6): 8.32 (1 H, t, J 8.9 Hz, Ar-H), 8.23 (2 H, d, J 8.7 Hz, Ar-H), 7.96 (1 H, d, J 8.7 Hz, Ar-H), 7.78 (1 H, dd, J 12.0 Hz, 2.4 Hz, Ar-H), 7.48 (3 H, m, Ar-H), 6.71 (1 H, d, J 2.3 Hz, Ar-H), 6.67 (1 H, dd, J 8.7 Hz, 2.3 Hz, Ar-H), 4.10 (2 H, t, J 6.2 Hz, O-$\underline{CH_2}$-CH_2-), 3.87 (3 H, s, O-$\underline{CH_3}$), 1.72 (2 H, tt, J 6.2 Hz, 6.8 Hz, O-CH_2-$\underline{CH_2}$-CH_2-), 1.47 (2 H, tq, J 6.8 Hz, 7.3 Hz, O-CH_2-CH_2-$\underline{CH_2}$-CH_3), 0.90 (3 H, t, J 7.3 Hz, O-CH_2-CH_2-CH_2-$\underline{CH_3}$)

δ_F/ppm (376 MHz, DMSO-d_6): -115.37

δ_C/ppm (100 MHz, DMSO-d_6): 165.42, 163.43, 163.13, 161.72, 156.99, 156.09, 155.99, 154.38, 135.23, 135.16, 134.50, 132.29, 128.01, 127.99, 125.76, 123.19, 119.71, 119.67, 113.42, 113.18, 110.31, 106.31, 100.06, 68.52, 56.19, 31.09, 19.13, 14.08

MS = $[M+Na]^+$: Calculated for $C_{25}H_{22}NO_8FNa$: 506.1227. Found: 516.1226. Difference: 0.2 ppm

6.4 4-[(3-Fluoro-4-nitrophenoxy)carbonyl]phenyl 4-methoxy-2-pentoxybenzoate (6-5)

Yield: 0.088 g, 21.1 %. RF: 0.403 (100 % dichloromethane).

T_{CrI} 101 °C T_{N_FI} (56 °C)

ν_{max}/cm^{-1}: 3059, 2945, 2874, 1750, 1743, 1602, 1577, 1527, 1507, 1482, 1471, 1444, 1427, 1395, 1349, 1335, 1299, 1271, 1252, 1233, 1204, 1180, 1158, 1129, 1093, 1050, 1001, 967, 959, 893, 872, 841, 820, 806, 757, 746, 735, 720, 690, 679, 632, 608, 580, 540, 508, 459, 415

δ_H/ppm (400 MHz, DMSO-d_6): 8.32 (1 H, t, J 8.9 Hz, Ar-H), 8.24 (2 H, d, J 8.8 Hz, Ar-H), 7.95 (1 H, d, J 8.7 Hz, Ar-H), 7.79 (1 H, dd, J 12.0 Hz, 2.4 Hz, Ar-H), 7.47 (3 H, m, Ar-H), 6.71 (1 H, d, J 2.3 Hz, Ar-H), 6.67 (1 H, dd, J 8.7 Hz, 2.3 Hz, Ar-H), 4.09 (2 H, t, J 6.2 Hz, O-$\underline{CH_2}$-CH_2-), 3.87 (3 H, s, O-$\underline{CH_3}$), 1.73 (2 H, tt, J 6.2 Hz, 6.8 Hz, O-CH_2-$\underline{CH_2}$-CH_2-), 1.43 (2 H, tt, J 6.8 Hz, 7.2 Hz, O-CH_2-CH_2-$\underline{CH_2}$-CH_2-), 1.31 (2 H, sept, J 7.2 Hz, O-CH_2-CH_2-CH_2-$\underline{CH_2}$-CH_3), 0.83 (3 H, t, J 7.2 Hz, O-CH_2-CH_2-CH_2-CH_2-$\underline{CH_3}$)

δ_F/ppm (376 MHz, DMSO-d_6): -115.37

δ_C/ppm (100 MHz, DMSO-d_6): 165.42, 163.44, 163.23, 161.68, 156.99, 156.09, 155.99, 154.38, 135.23, 135.16, 134.52, 132.28, 128.01, 127.99, 125.76, 123.19, 119.71, 119.68, 113.43, 113.19, 110.33, 106.32, 100.06, 68.82, 56.19, 28.71, 28.12, 22.24, 14.36

MS = $[M+Na]^+$: Calculated for $C_{26}H_{24}NO_8FNa$: 520.1384. Found: 520.1362. Difference: 4.2 ppm

6.5 4-[(3-Fluoro-4-nitrophenoxy)carbonyl]phenyl 2-hexyloxy-4-methoxybenzoate (6-6)

Yield: 0.059 g, 14.4 %. RF: 0.361 (20 % ethyl acetate: 80 % 40:60 petroleum ether).

T_{CrI} 77 °C T_{N_FI} (48 °C)

ν_{max}/cm^{-1}: 2950, 2875, 1759, 1746, 1601, 1526, 1507, 1481, 1470, 1444, 1414, 1396, 1349, 1331, 1298, 1271, 1250, 1234, 1204, 1181, 1153, 1129, 1092, 1048, 1035, 1005, 997, 966, 955, 892, 871, 853, 840, 822, 806, 756, 745, 734, 717, 689, 679, 631, 608, 580, 540, 508, 481, 457, 410

δ_H/ppm (400 MHz, DMSO-d_6): 8.33 (1 H, t, J 8.9 Hz, Ar-H), 8.24 (2 H, d, J 8.6 Hz, Ar-H), 7.96 (1 H, d, J 8.7 Hz, Ar-H), 7.80 (1 H, dd, J 12.0 Hz, 2.4 Hz, Ar-H), 7.49 (3 H, m, Ar-H), 6.70 (1 H, d, J 2.3 Hz, Ar-H), 6.66 (1 H, dd, J 8.7 Hz, J 2.3 Hz, Ar-H), 4.10 (2 H, t, J 6.2 Hz, O-$\underline{CH_2}$-CH_2-), 3.88 (3 H, s, O-$\underline{CH_3}$), 1.73 (2 H, tt, J 6.2 Hz, 6.8 Hz, O-CH_2-$\underline{CH_2}$-CH_2-), 1.44 (2 H, quin, J 6.8 Hz, O-CH_2-

CH_2-$\underline{CH_2}$-CH_2-), 1.24 (4 H, m, O-CH_2-CH_2-CH_2-$\underline{CH_2\text{-}CH_2}$-$CH_3$), 0.82 (3 H, t, J 6.8 Hz, O-CH_2-CH_2-CH_2-CH_2-CH_2-$\underline{CH_3}$)
δ_F/ppm (376 MHz, DMSO-d_6): -115.37
δ_C/ppm (100 MHz, DMSO-d_6): 165.41, 163.44, 163.24, 161.67, 156.99, 156.10, 155.98, 154.38, 135.24, 135.17, 134.51, 132.28, 128.00, 127.99, 125.76, 123.18, 119.72, 119.68, 113.43, 113.19, 110.35, 106.33, 100.07, 68.84, 56.20, 31.35, 29.00, 25.59, 22.51, 14.31
MS = $[M+Na]^+$: Calculated for $C_{27}H_{26}NO_8FNa$: 534.1540. Found: 534.1560. Difference: 3.7 ppm

6.6 4-[(3-Fluoro-4-nitrophenoxy)carbonyl]phenyl 2-heptyloxy-4-methoxybenzoate (6-7)
Yield: 0.040 g, 9.81 %. RF: 0.447 (100 % dichloromethane).
T_{CrI} 93 °C T_{N_FI} (47 °C)
ν_{max}/cm^{-1}: 2923, 2857, 1732, 1602, 1572, 1534, 1504, 1486, 1469, 1441, 1430, 1418, 1389, 1347, 1297, 1241, 1198, 1167, 1139, 1093, 1047, 1021, 1006, 966, 893, 840, 804, 757, 726, 691, 670, 630, 616 ,600, 546, 537, 506, 474, 418
δ_H/ppm (400 MHz, DMSO-d_6): 8.32 (1 H, t, J 8.8 Hz, Ar-H), 8.23 (2 H, d, J 8.6 Hz, Ar-H), 7.94 (1 H, d, J 8.7 Hz, Ar-H), 7.78 (1 H, dd, J 12.0 Hz, 2.4 Hz, Ar-H), 7.47 (3 H, m, Ar-H), 6.70 (1 H, d, J 2.2 Hz, Ar-H), 6.67 (1 H, dd, J 8.7 Hz, 2.2 Hz, Ar-H), 4.09 (2 H, t, J 6.2 Hz, O-$\underline{CH_2}$-CH_2-), 3.87 (3 H, s, O-$\underline{CH_3}$), 1.72 (2 H, tt, J 6.2 Hz, 6.9 Hz, O-CH_2-$\underline{CH_2}$-CH_2-), 1.43 (2 H, m, O-CH_2-CH_2-$\underline{CH_2}$-CH_2-), 1.22 (6 H, m, O-CH_2-CH_2-CH_2-$\underline{CH_2\text{-}CH_2\text{-}CH_2}$-$CH_3$), 0.81 (3 H, t, J 7.0 Hz, O-CH_2-CH_2-CH_2-CH_2-CH_2-$\underline{CH_3}$)
δ_F/ppm (376 MHz, DMSO-d_6): -115.35
δ_C/ppm (100 MHz, DMSO-d_6): 165.41, 163.42, 163.27, 161.66, 156.99, 156.10, 155.97, 154.38, 135.23, 135.16, 134.50, 132.26, 128.02, 128.00, 125.73, 123.16, 119.68, 119.64, 113.40, 113.16, 110.36, 106.31, 100.06, 68.83, 56.18, 31.69, 29.06, 28.83, 25.89, 22.47, 14.38
MS = $[M+Na]^+$: Calculated for $C_{28}H_{28}NO_8FNa$: 548.1697. Found: 548.1700. Difference: 0.5 ppm

6.7 4-[(3-Fluoro-4-nitrophenoxy)carbonyl]phenyl 4-methoxy-2-octyloxybenzoate (6-8)
Yield: 0.122 g, 30.2 %. RF: 0.529 (100 % dichloromethane).
T_{CrI} 111 °C T_{N_FI} (47 °C)
ν_{max}/cm^{-1}: 2925, 2856, 1754, 1719, 1600, 1571, 1522, 1508, 1489, 1466, 1440, 1382, 1349, 1297, 1240, 1203, 1168, 1152, 1141, 1129, 1094, 1054, 1030, 1014, 969, 901, 890, 833, 811, 750, 689, 667, 644, 630, 613, 587, 534, 505, 462
δ_H/ppm (400 MHz, DMSO-d_6): 8.32 (1 H, t, J 8.9 Hz, Ar-H), 8.23 (2 H, d, J 8.7 Hz, Ar-H), 7.94 (1 H, d, J 8.7 Hz, Ar-H), 7.78 (1 H, dd, J 12.0 Hz, 2.4 Hz, Ar-H), 7.48 (3 H, m, Ar-H), 6.70 (1 H, d, J 2.3 Hz, Ar-H), 6.67 (1 H, dd, J 8.7 Hz, 2.3 Hz, Ar-H), 4.09 (2 H, t, J 6.2 Hz, O-$\underline{CH_2}$-CH_2-), 3.87 (3 H, s, O-$\underline{CH_3}$), 1.72 (2 H, tt, J 6.2 Hz, 6.8 Hz, O-CH_2-$\underline{CH_2}$-CH_2-), 1.43 (2 H, tt, J 6.8 Hz, 7.1 Hz, O-CH_2-CH_2-$\underline{CH_2}$-CH_2-), 1.20 (8 H, m, O-CH_2-CH_2-CH_2-$\underline{CH_2\text{-}CH_2\text{-}CH_2\text{-}CH_2}$-$CH_3$), 0.81 (3 H, t, J 7.1 Hz, O-CH_2-CH_2-CH_2-CH_2-CH_2-CH_2-CH_2-$\underline{CH_3}$)
δ_F/ppm (376 MHz, DMSO-d_6): -115.34
δ_C/ppm (100 MHz, DMSO-d_6): 165.41, 163.42, 163.30, 161.65, 157.00, 156.11, 156.07, 155.96, 154.38, 135.24, 135.17, 134.50, 132.27, 128.04, 128.01, 125.73, 123.16, 119.67, 119.64, 113.39, 113.16, 110.37, 106.33, 100.07, 68.84, 56.19, 31.65, 29.14, 29.06, 25.96, 22.54, 14.38
MS = $[M+Na]^+$: Calculated for $C_{29}H_{30}NO_8FNa$: 562.1835. Found: 562.1873. Difference: 3.6 ppm

6.8 4-[(3-Fluoro-4-nitrophenoxy)carbonyl]phenyl 4-methoxy-2-nonyloxybenzoate (6-9)
Yield: 0.070 g, 17.5 %. RF: 0.474 (100 % dichloromethane).
T_{CrI} 105 °C T_{N_FI} (46 °C)

v_{max}/cm^{-1}: 2924, 2855, 1754, 1717, 1600, 1572, 1522, 1508, 1488, 1466, 1441, 1412, 1386, 1350, 1271, 1248, 1204, 1169, 1152, 1141, 1129, 1095, 1054, 1030, 1014, 969, 903, 888, 844, 834, 811, 750, 689, 668, 645, 632, 611, 587, 575, 535, 505, 463, 407

δ_H/ppm (400 MHz, DMSO-d_6): 8.32 (1 H, t, J 8.9 Hz, Ar-H), 8.23 (2 H, d, J 8.8 Hz, Ar-H), 7.94 (1 H, d, J 8.7 Hz, Ar-H), 7.77 (1 H, dd, J 12.0 Hz, 2.4 Hz, Ar-H), 7.47 (3 H, m, Ar-H), 6.70 (1 H, d, J 2.3 Hz, Ar-H), 6.67 (1 H, dd, J 8.7 Hz, 2.3 Hz, Ar-H), 4.08 (2 H, t, J 6.2 Hz, O-$\underline{CH_2}$-CH_2-), 3.87 (3 H, s, O-$\underline{CH_3}$), 1.72 (2 H, tt, J 6.2 Hz, 7.0 Hz, O-CH_2-$\underline{CH_2}$-CH_2-), 1.42 (2 H, quin, J 7.0 Hz, O-CH_2-CH_2-$\underline{CH_2}$-CH_2-), 1.19 (10 H, m, O-CH_2-CH_2-CH_2-$\underline{CH_2\text{-}CH_2\text{-}CH_2\text{-}CH_2\text{-}CH_2}$-$CH_3$), 0.81 (3 H, t, J 7.0 Hz, O-CH_2-CH_2-CH_2-CH_2-CH_2-CH_2-CH_2-CH_2-$\underline{CH_3}$)

δ_F/ppm (376 MHz, DMSO-d_6): -115.34

δ_C/ppm (100 MHz, DMSO-d_6): 165.41, 163.41, 163.31, 161.65, 157.00, 156.11, 156.07, 155.96, 154.38, 135.23, 135.16, 134.51, 132.26, 128.03, 128.01, 125.72, 123.16, 119.65, 119.61, 113.37, 113.13, 110.36, 106.32, 100.06, 68.83, 56.18, 31.74, 29.43, 29.18, 29.07, 25.95, 22.53, 14.39

MS = $[M+H]^+$: Calculated for $C_{30}H_{33}NO_8F$: 554.2190. Found: 554.2211. Difference: 3.8 ppm

Differential Scanning Calorimetry Data for New Compounds

5-7

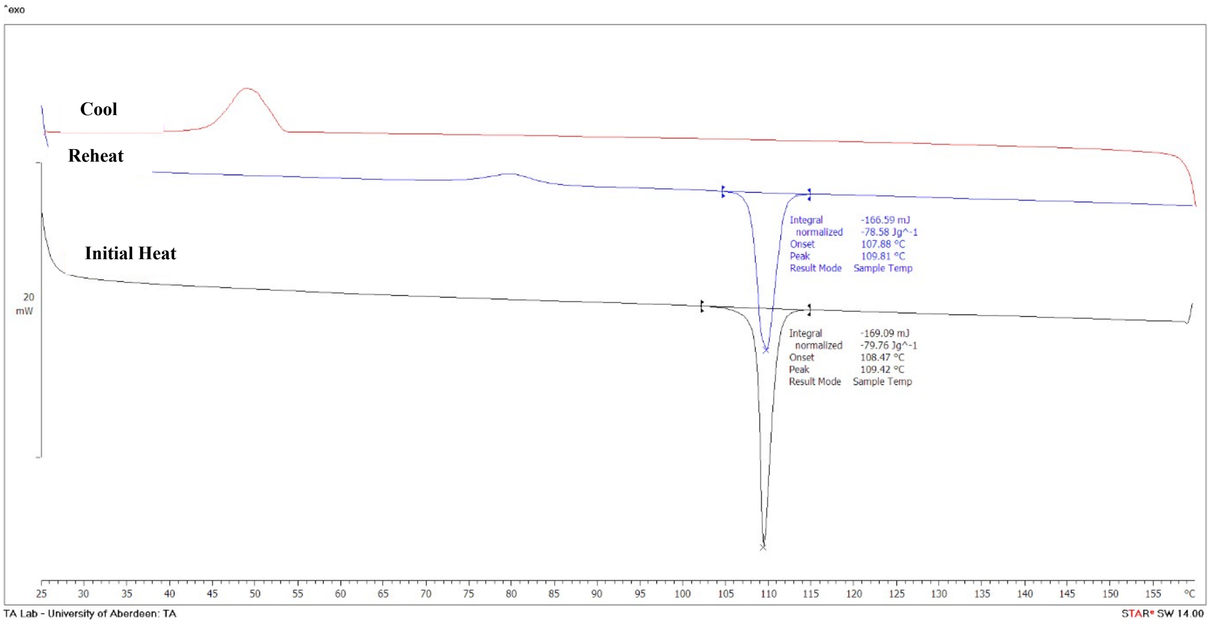

5-8

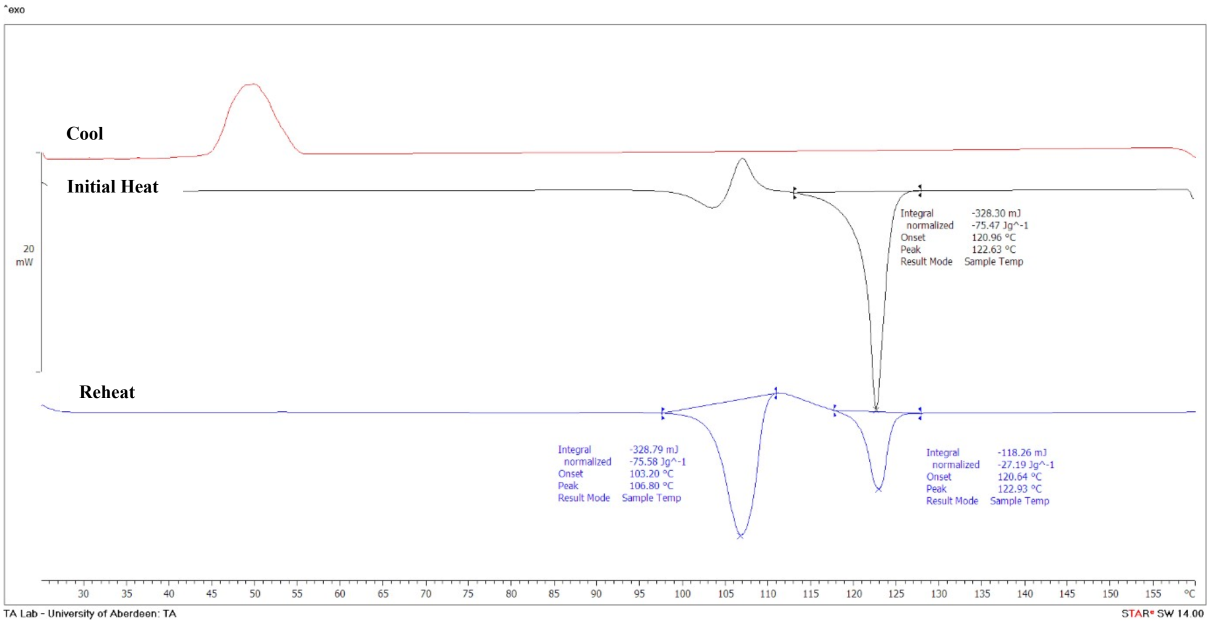

5-9

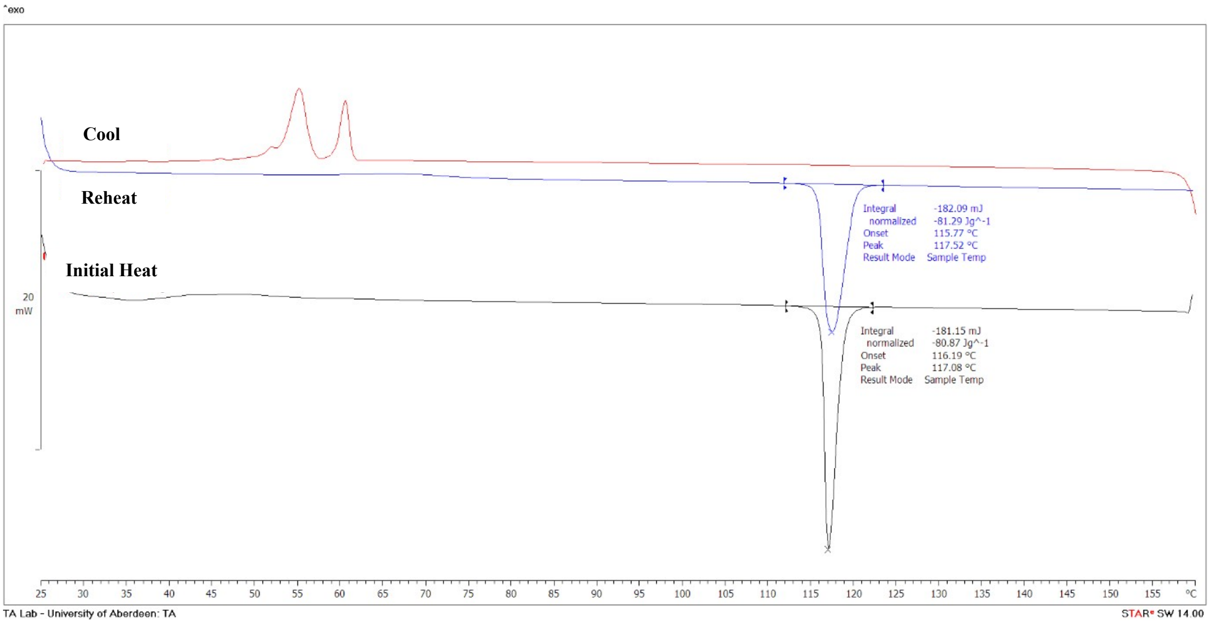
^exo
Cool
Reheat
Initial Heat
20
mW
Integral -182.09 mJ
normalized -81.29 Jg^-1
Onset 115.77 °C
Peak 117.52 °C
Result Mode Sample Temp
Integral -181.15 mJ
normalized -80.87 Jg^-1
Onset 116.19 °C
Peak 117.08 °C
Result Mode Sample Temp
25 30 35 40 45 50 55 60 65 70 75 80 85 90 95 100 105 110 115 120 125 130 135 140 145 150 155 °C
TA Lab - University of Aberdeen: TA
STAR® SW 14.00

6-2

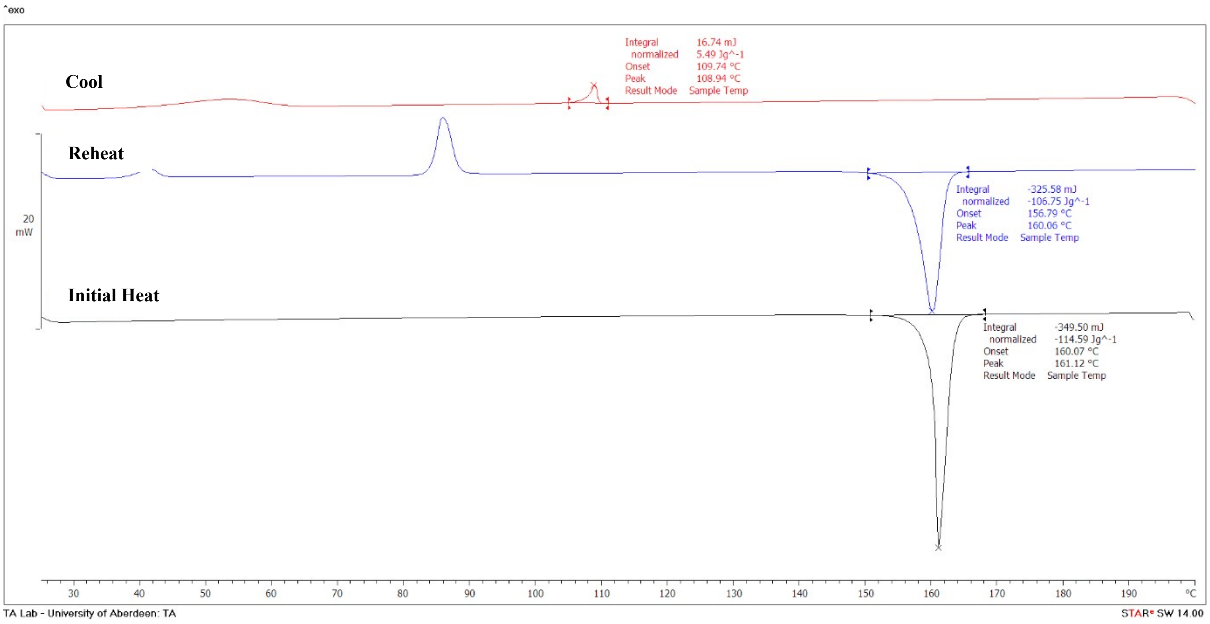
^exo
Integral 16.74 mJ
normalized 5.49 Jg^-1
Onset 109.74 °C
Peak 108.94 °C
Result Mode Sample Temp
Cool
Reheat
20
mW
Integral -325.58 mJ
normalized -106.75 Jg^-1
Onset 156.79 °C
Peak 160.06 °C
Result Mode Sample Temp
Initial Heat
Integral -349.50 mJ
normalized -114.59 Jg^-1
Onset 160.07 °C
Peak 161.12 °C
Result Mode Sample Temp
30 40 50 60 70 80 90 100 110 120 130 140 150 160 170 180 190 °C
TA Lab - University of Aberdeen: TA
STAR® SW 14.00

6-3

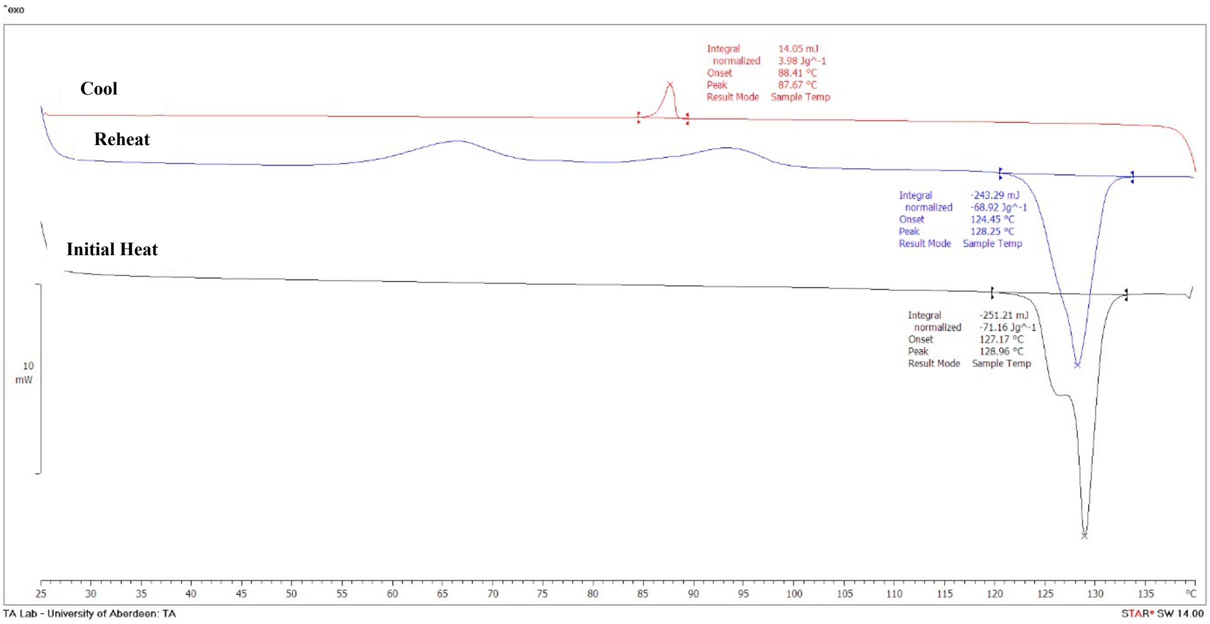
^exo
Cool
Reheat
Initial Heat
Integral 14.05 mJ
normalized 3.98 Jg^-1
Onset 88.41 °C
Peak 87.67 °C
Result Mode Sample Temp
Integral -243.29 mJ
normalized -68.92 Jg^-1
Onset 124.45 °C
Peak 128.25 °C
Result Mode Sample Temp
Integral -251.21 mJ
normalized -71.16 Jg^-1
Onset 127.17 °C
Peak 128.96 °C
Result Mode Sample Temp
10 mW
25 30 35 40 45 50 55 60 65 70 75 80 85 90 95 100 105 110 115 120 125 130 135 °C
TA Lab - University of Aberdeen: TA
STAR® SW 14.00

6-4

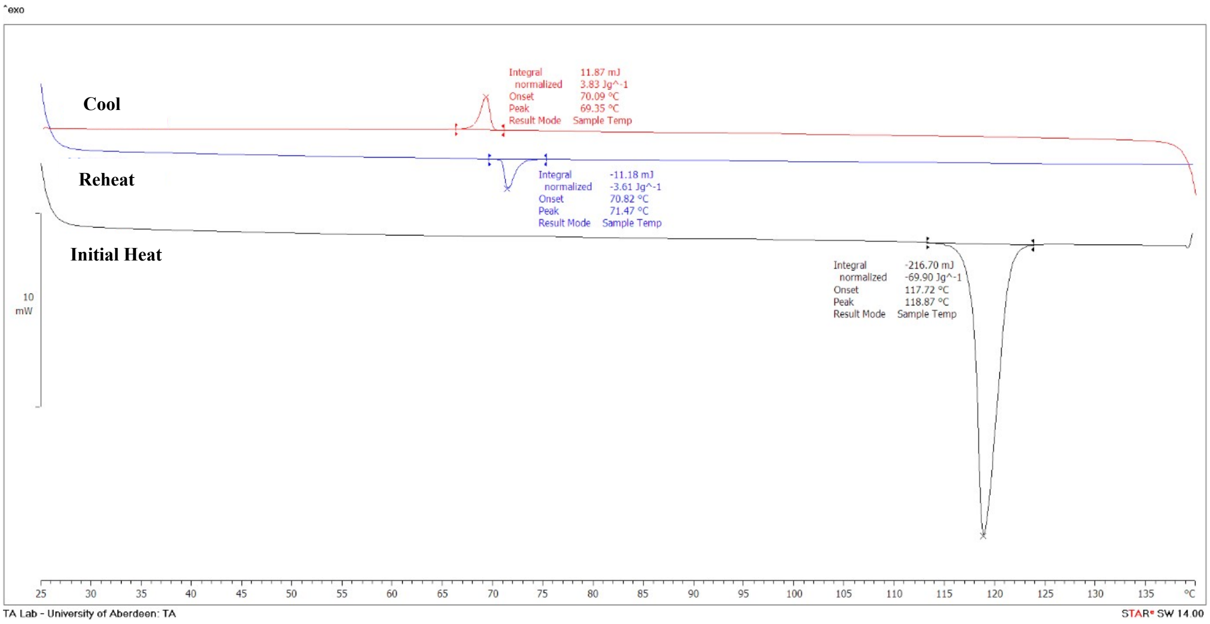
^exo
Cool
Reheat
Initial Heat
Integral 11.87 mJ
normalized 3.83 Jg^-1
Onset 70.09 °C
Peak 69.35 °C
Result Mode Sample Temp
Integral -11.18 mJ
normalized -3.61 Jg^-1
Onset 70.82 °C
Peak 71.47 °C
Result Mode Sample Temp
Integral -216.70 mJ
normalized -69.90 Jg^-1
Onset 117.72 °C
Peak 118.87 °C
Result Mode Sample Temp
10 mW
25 30 35 40 45 50 55 60 65 70 75 80 85 90 95 100 105 110 115 120 125 130 135 °C
TA Lab - University of Aberdeen: TA
STAR® SW 14.00

6-5

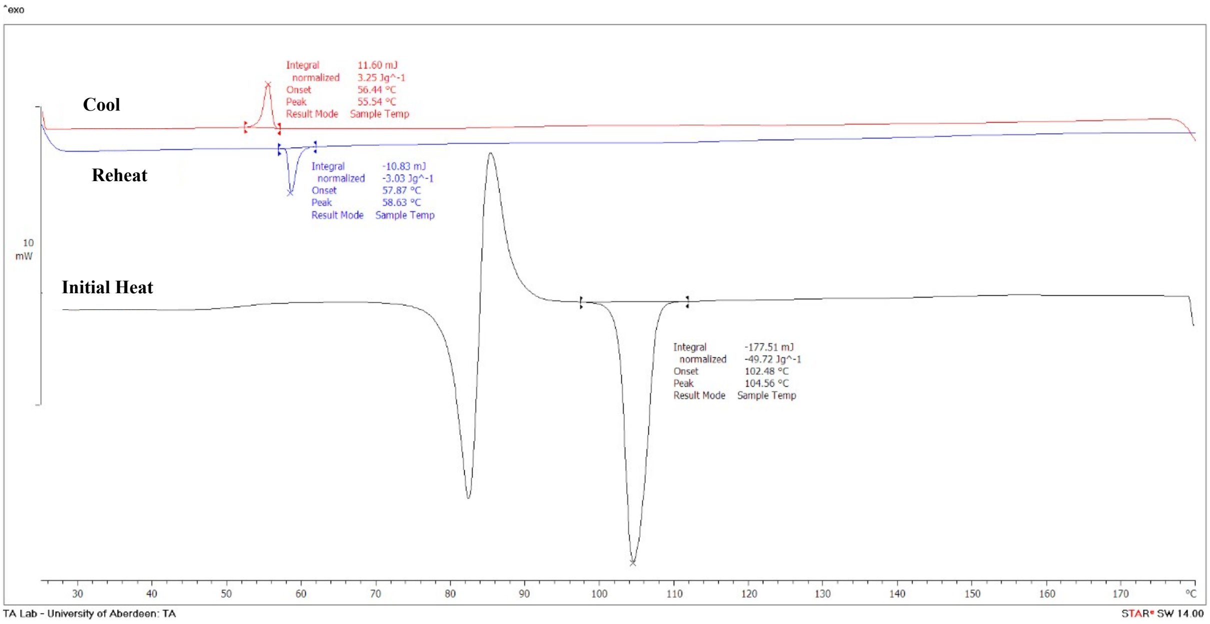
^exo
Cool
Integral 11.60 mJ
normalized 3.25 Jg^-1
Onset 56.44 °C
Peak 55.54 °C
Result Mode Sample Temp
Reheat
Integral -10.83 mJ
normalized -3.03 Jg^-1
Onset 57.87 °C
Peak 58.63 °C
Result Mode Sample Temp
10 mW
Initial Heat
Integral -177.51 mJ
normalized -49.72 Jg^-1
Onset 102.48 °C
Peak 104.56 °C
Result Mode Sample Temp
30 40 50 60 70 80 90 100 110 120 130 140 150 160 170 °C
TA Lab - University of Aberdeen: TA
STAR^e SW 14.00

6-7

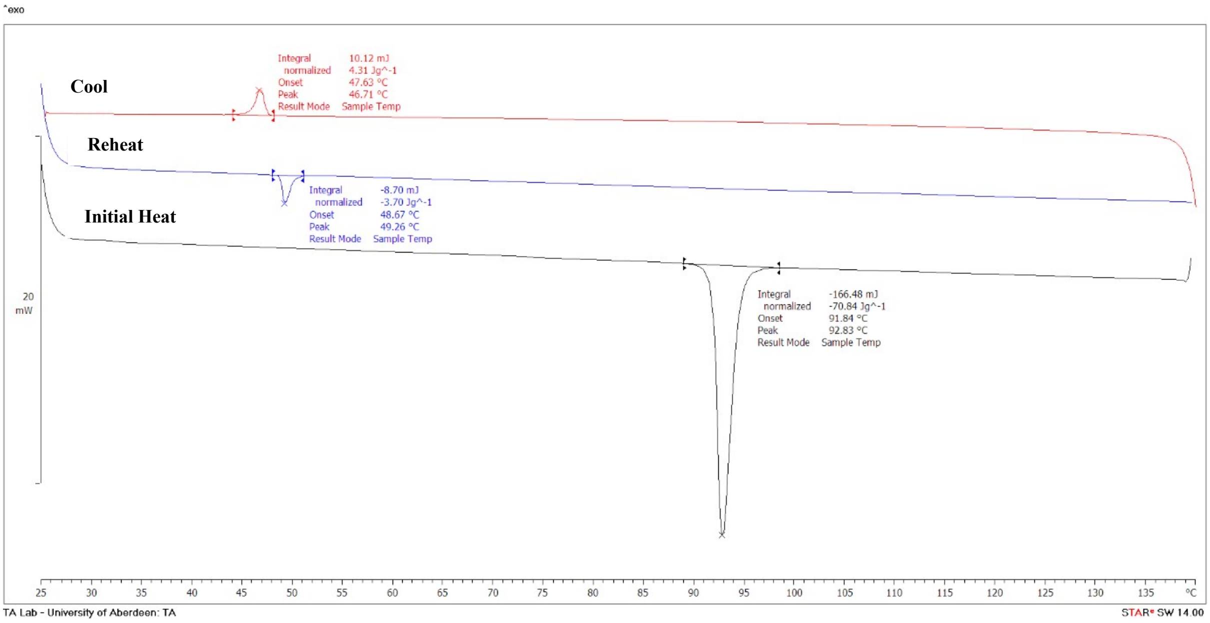
^exo
Cool
Integral 10.12 mJ
normalized 4.31 Jg^-1
Onset 47.63 °C
Peak 46.71 °C
Result Mode Sample Temp
Reheat
Integral -8.70 mJ
normalized -3.70 Jg^-1
Onset 48.67 °C
Peak 49.26 °C
Result Mode Sample Temp
Initial Heat
20 mW
Integral -166.48 mJ
normalized -70.84 Jg^-1
Onset 91.84 °C
Peak 92.83 °C
Result Mode Sample Temp
25 30 35 40 45 50 55 60 65 70 75 80 85 90 95 100 105 110 115 120 125 130 135 °C
TA Lab - University of Aberdeen: TA
STAR^e SW 14.00

6-8

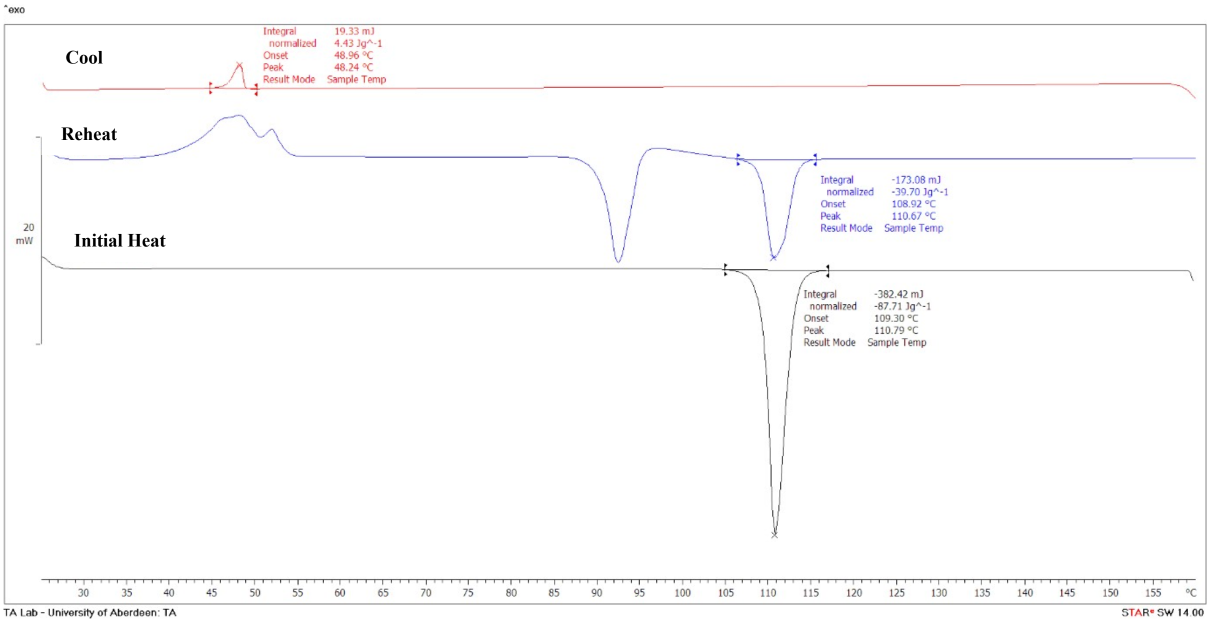
^exo
Cool
Integral 19.33 mJ
normalized 4.43 Jg^-1
Onset 48.96 °C
Peak 48.24 °C
Result Mode Sample Temp
Reheat
Integral -173.08 mJ
normalized -39.70 Jg^-1
Onset 108.92 °C
Peak 110.67 °C
Result Mode Sample Temp
20 mW
Initial Heat
Integral -382.42 mJ
normalized -87.71 Jg^-1
Onset 109.30 °C
Peak 110.79 °C
Result Mode Sample Temp
30 35 40 45 50 55 60 65 70 75 80 85 90 95 100 105 110 115 120 125 130 135 140 145 150 155 °C
TA Lab - University of Aberdeen: TA
STAR® SW 14.00

6-9

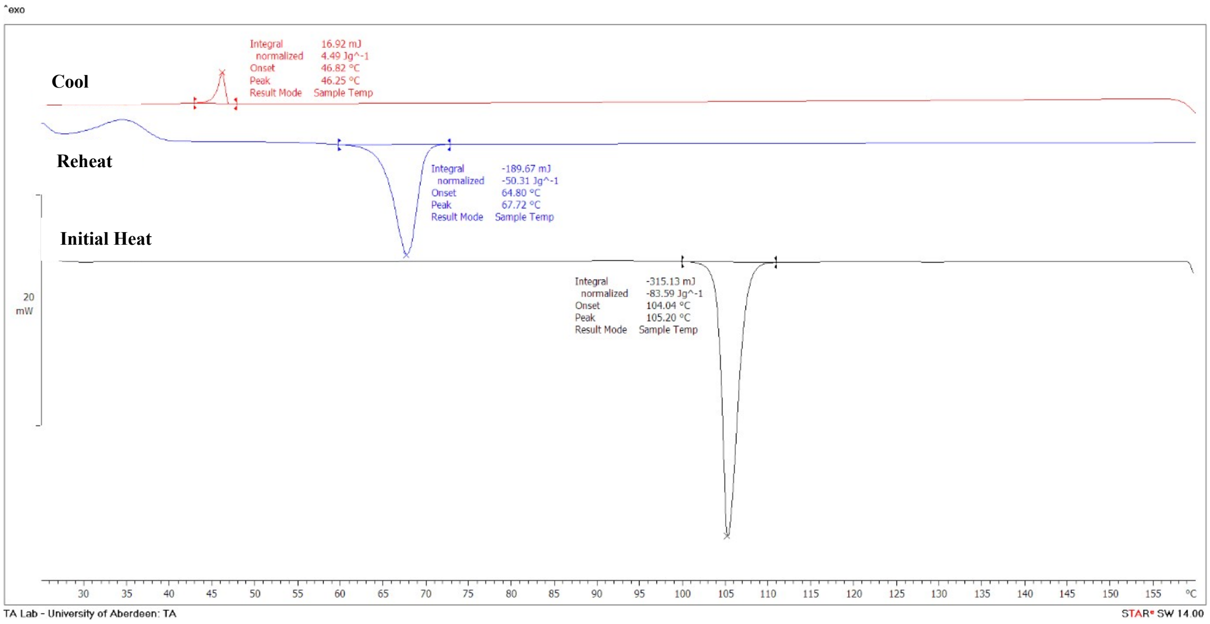
^exo
Integral 16.92 mJ
normalized 4.49 Jg^-1
Onset 46.82 °C
Peak 46.25 °C
Result Mode Sample Temp
Cool
Reheat
Integral -189.67 mJ
normalized -50.31 Jg^-1
Onset 64.80 °C
Peak 67.72 °C
Result Mode Sample Temp
Initial Heat
20 mW
Integral -315.13 mJ
normalized -83.59 Jg^-1
Onset 104.04 °C
Peak 105.20 °C
Result Mode Sample Temp
30 35 40 45 50 55 60 65 70 75 80 85 90 95 100 105 110 115 120 125 130 135 140 145 150 155 °C
TA Lab - University of Aberdeen: TA
STAR® SW 14.00

Polarised Optical Microscope Textures for New Compounds

5-7

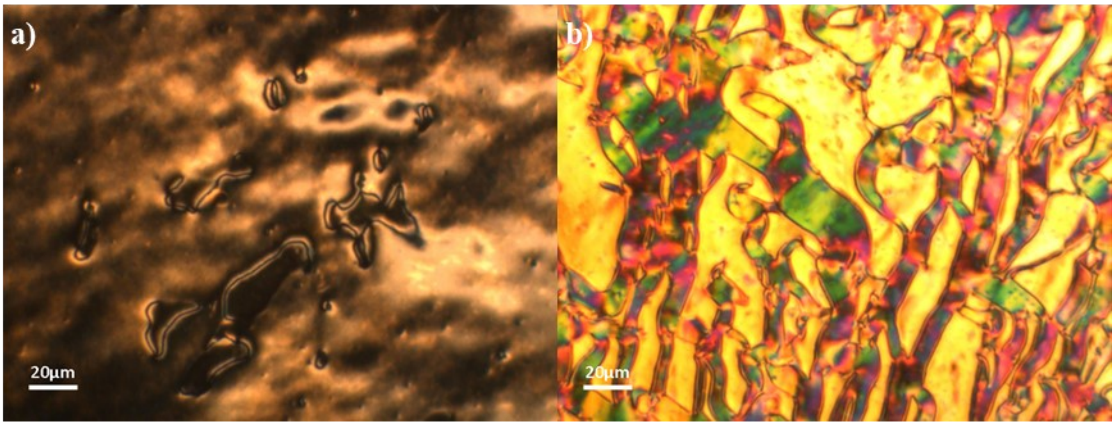

Figure S1. (a) schlieren texture of the N phase between untreated glass slides (T = 46 °C); (b) banded texture of the N_F phase between untreated glass slides (T = 28 °C).

5-8

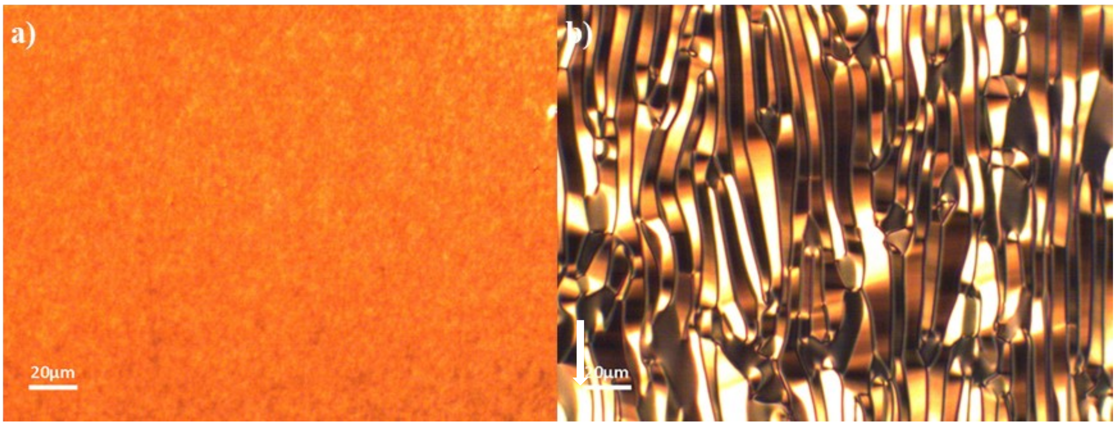

Figure S2. (a) uniform texture of the N phase in a planar aligned cell (T = 46 °C); (b) banded texture of the N_F phase in a planar aligned cell (T = 41 °C). The arrow indicates the alignment direction.

5-9

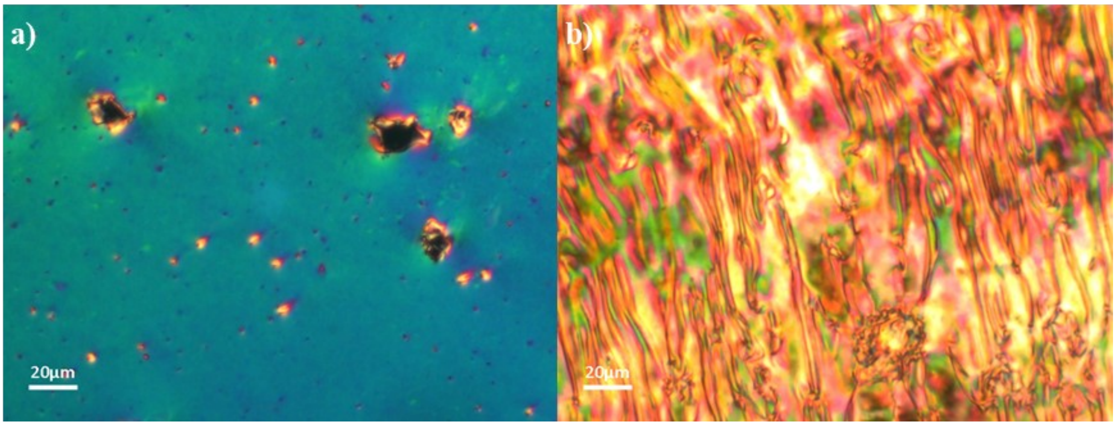

Figure S3. (a) uniform texture of the N phase between untreated glass slides (T = 45 °C); (b) domains of differing birefringence with boundaries in the N_F phase between untreated glass slides (T = 24 °C).

6-3

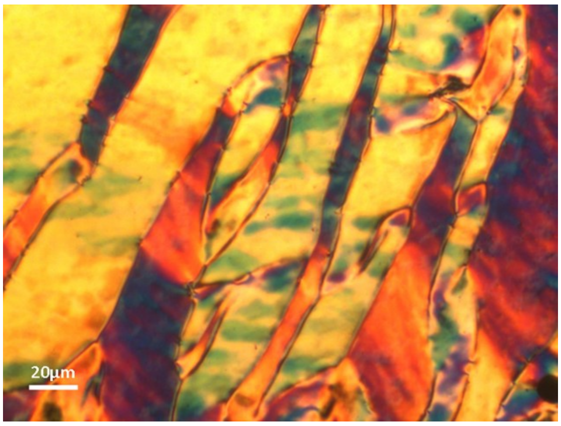

Figure S4. Banded texture of the N_F phase between untreated glass slides (T = 53 °C).

6-4

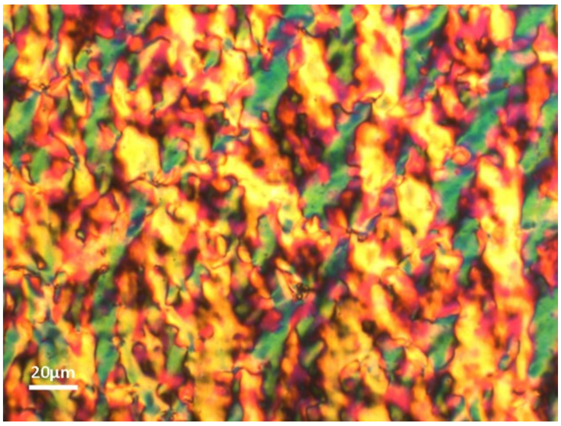

Figure S5. Domains of differing birefringence with boundaries in the N_F phase between untreated glass slides (T = 51 °C).

6-5

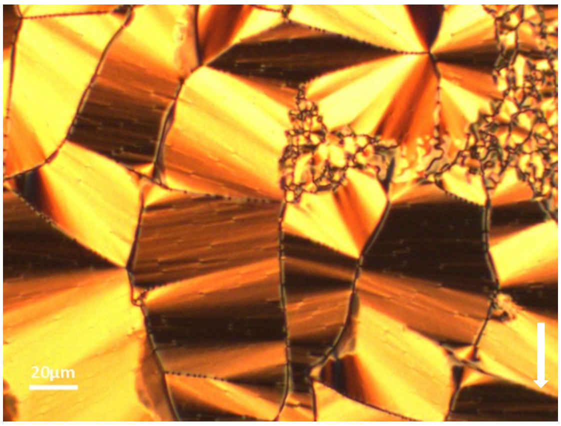

Figure S6. Banded texture of the N_F phase in a planar aligned cell (T = 47 °C). The arrow indicates the alignment direction.

6-7

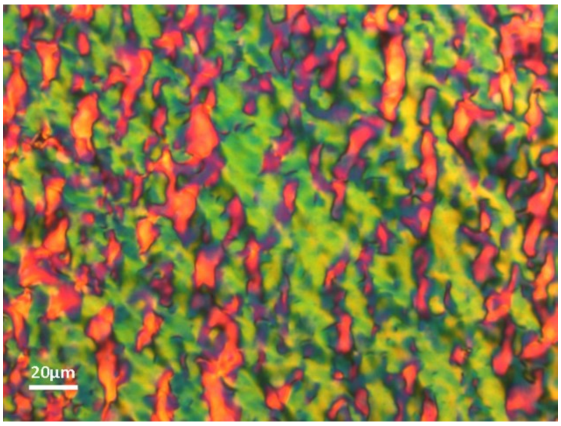

Figure S7. Domains of differing birefringence with boundaries in the N_F phase between untreated glass slides (T = 27 °C).

6-8

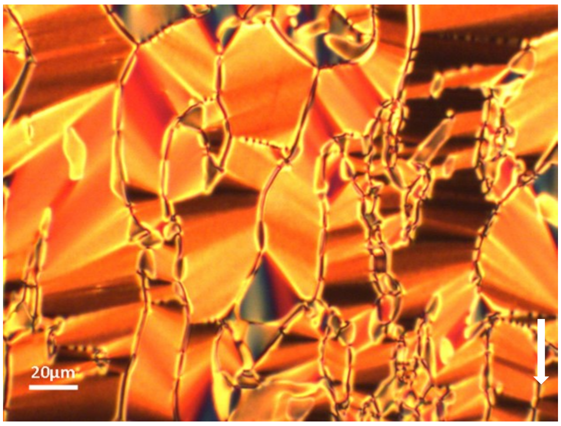

Figure S8. Banded texture of the N_F phase in a planar aligned cell (T = 44 °C). The arrow indicates the alignment direction.

6-9

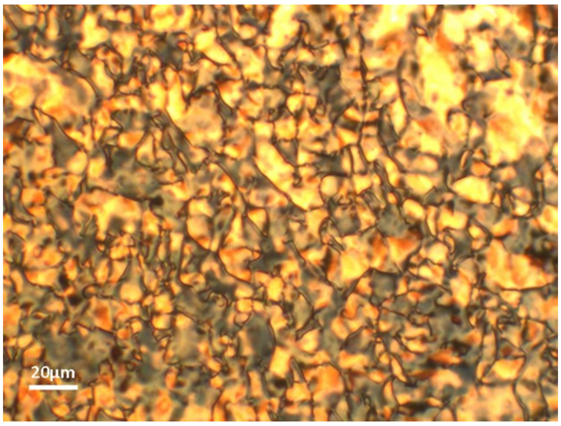

Figure S9. Banded texture of the N_F phase between untreated glass slides (T = 26 °C).

Materials and Methods

Reagents

All reagents and solvents that were available commercially were purchased from Sigma Aldrich, Fisher Scientific or Fluorochem and were used without further purification unless otherwise stated.

Thin Layer Chromatography

Reactions were monitored using thin layer chromatography, and the appropriate solvent system, using aluminium-backed plates with a coating of Merck Kieselgel 60 F254 silica which were purchased from Merck KGaA. The spots on the plate were visualised by UV light (254 nm).

Column Chromatography

For normal phase column chromatography, the separations were carried out using silica gel grade 60 Å, 40-63 µm particle size, purchased from Fluorochem and using an appropriate solvent system.

Structure Characterisation

All final products and intermediates that were synthesised were characterised using ^{1}H NMR, ^{19}F NMR, ^{13}C NMR and infrared spectroscopies. The NMR spectra were recorded on a 400 MHz Bruker Avance III HD NMR spectrometer. The infrared spectra were recorded on a Thermal Scientific Nicolet IR100 FTIR spectrometer with an ATR diamond cell.

Purity Analysis

In order to determine the purity of the final products, high-resolution mass spectrometry was carried out using a Waters XEVO G2 Q-Tof mass spectrometer by Dr. Morag Douglas at the University of Aberdeen.
